# Cardiovascular risk associated with the use of glitazones, metformin and sufonylureas: meta-analysis of published observational studies

**DOI:** 10.1186/s12872-016-0187-5

**Published:** 2016-01-15

**Authors:** Manel Pladevall, Nuria Riera-Guardia, Andrea V Margulis, Cristina Varas-Lorenzo, Brian Calingaert, Susana Perez-Gutthann

**Affiliations:** RTI Health Solutions, Trav. Gracia 56 Atico 1 08006, Barcelona, Spain; The Center for Health Policy and Health Services Research, Henry Ford Health System, Detroit, Michigan USA; RTI Health Solutions, Research Triangle Park, NC USA

**Keywords:** Blood glucose–lowering drugs, Type 2 diabetes mellitus, Stroke, AMI, Meta-analysis, Cardiovascular safety, Pharmacoepidemiology, Observational studies

## Abstract

**Background:**

The results of observational studies evaluating and comparing the cardiovascular safety of glitazones, metformin and sufonylureas are inconsistent.To conduct and evaluate heterogeneity in a meta-analysis of observational studies on the risk of acute myocardial infarction (AMI) or stroke in patients with type 2 diabetes using non-insulin blood glucose–lowering drugs (NIBGLD).

**Methods:**

We systematically identified and reviewed studies evaluating NIBGLD in patients with type 2 diabetes indexed in Medline, Embase, or the Cochrane Library that met prespecified criteria. The quality of included studies was assessed with the RTI item bank. Results were combined using fixed- and random-effects models, and the Higgins *I*^*2*^ statistic was used to evaluate heterogeneity. Sensitivity analyses by study quality were conducted.

**Results:**

The summary relative risk (sRR) (95 % CI) of AMI for rosiglitazone versus pioglitazone was 1.13 (1.04–1.24) [*I*^*2*^ = 55 %]. In the sensitivity analysis, heterogeneity was reduced [*I*^*2*^ = 16 %]. The sRR (95 % CI) of stroke for rosiglitazone versus pioglitazone was 1.18 (1.02–1.36) [*I*^*2*^ = 42 %]. There was strong evidence of heterogeneity related to study quality in the comparisons of rosiglitazone versus metformin and rosiglitazone versus sulfonylureas (*I*^*2*^ ≥ 70 %). The sRR (95 % CI) of AMI for sulfonylurea versus metformin was 1.24 (1.14–1.34) [*I*^*2*^ = 41 %] and for pioglitazone versus metformin was 1.02 (0.75–1.38) [*I*^*2*^ = 17 %]. Sensitivity analyses decreased heterogeneity in most comparisons.

**Conclusion/interpretation:**

Sulfonylureas increased the risk of AMI by 24 % compared with metformin; an imprecise point estimate indicated no difference in risk of AMI when comparing pioglitazone with metformin. The presence of heterogeneity precluded any conclusions on the other comparisons. The quality assessment was valuable in identifying methodological problems in the individual studies and for analysing potential sources of heterogeneity.

**Electronic supplementary material:**

The online version of this article (doi:10.1186/s12872-016-0187-5) contains supplementary material, which is available to authorized users.

## Background

Globally, about 1 billion people are overweight or obese, which will cause an increase of epidemic proportions in the number of persons with type 2 diabetes and cardiovascular disease. In 2011, 366 million people had been diagnosed with diabetes, and the number is expected to rise to 552 million by 2030. Most people with diabetes live in the developing world, and this area will see the greatest increase over the next 19 years [1].

Type 2 diabetes mellitus (T2DM) is characterised by high blood glucose levels that cause eye, kidney, and nerve complications and an increased risk for cardiovascular disease. Most of the morbidity and mortality associated with diabetes is due to cardiovascular disease; diabetes is considered by some as a coronary heart disease risk–equivalent condition [2]. Although this concept is controversial [3], there is evidence that the risk equivalence might hold at least for women [4].

Controversy has surrounded the cardiovascular safety of some non-insulin blood glucose–lowering drugs (NIBGLD), particularly sulfonylureas and thiazolidinediones. Rosiglitazone was removed from the market in Europe and its use highly restricted in the United States of America (US) after review of safety evaluations from clinical trials suggested that its use increased the incidence of acute myocardial infarction (AMI) [5]. This and the premature termination of the ACCORD trial [6], after observing an increase in cardiovascular mortality in the group treated more aggressively, prompted the US Food and Drug Administration (FDA) to revise its policy for approving new drugs for T2DM; a two-step approach ensuring cardiovascular safety is now required. Evidence has led some to question rosiglitazone’s adverse cardiovascular safety profile [7–9]. Using observational data, compared with metformin, sulfonylureas have been associated with a 21 % increase in the risk of hospitalisation for AMI, stroke, or death [10].

Meta-analyses of clinical trials and observational studies reviewing the risk of AMI and/or stroke associated with the use of oral blood glucose–lowering drugs for T2DM have been published [11–18]. However, previous meta-analyses of observational studies have not investigated the heterogeneity present in results and methods of individual studies [11, 15, 16]. Heterogeneity, analysis, important for meta-analyses of observational studies [19], might explain inconsistencies among results of individual studies.

This research was part of the Safety Evaluation of Adverse Reactions in Diabetes (SAFEGUARD) project. This report summarises the results of a systematic review and meta-analysis of published observational studies on the risk of AMI and stroke in patients with T2DM receiving treatment with NIBGLD, analysis of the heterogeneity in study characteristics and results, and evaluation of study quality as an explanatory factor for statistical heterogeneity. We also provide results of drug comparisons not reported in previous meta-analyses. With the recent introduction in clinical practice of new classes of NIBGLD such as dipeptidyl peptidase-4 inhibitors, glucagon-like peptide-1 receptor agonists, and sodium-glucose cotransporter-2 inhibitors, it becomes crucial to evaluate the cardiovascular safety of older NIBGLD (e.g., glitazones, metformin and sufonylureas) before their use in clinical practice may change with the introduction of the newer agents, and newer clinical guidelines.

## Methods

We conducted a systematic literature search in Medline, Embase, and the Cochrane Library. The search was conducted on November 11, 2011 (see Additional file 1), and was limited to observational studies on humans (systematic reviews, meta-analyses, original articles), with no publication date or language restrictions for blood glucose–lowering drugs (except insulin); outcomes were AMI, stroke, and others reported elsewhere with details on the literature search, study selection, and data abstraction [20]. Studies including transient ischaemic attack in the definition of stroke were excluded. Studies including stable angina in the definition of AMI were excluded. We conducted an updated literature search in September 2014, and the impact of additional studies that were identified is reported in the discussion section.

We assessed the quality of each study included in the systematic review using two tools, the Newcastle-Ottawa Scale [21] and the RTI item bank (RTI-IB) on risk of bias and precision [22]. We used a version of the RTI-IB adapted to the research question with 31 items. It was applied independently by two researchers who discussed disagreements until reaching consensus, when necessary. Possible responses for most items denoted high, unclear, or low risk of bias. Additional details on the tool and how the quality assessment was performed are reported elsewhere [23].

Quantitative analysis was conducted using Review Manager software version 5.2.3 [24]. Additional details can be found elsewhere [20]. For each comparison with at least three independent point estimates available, summary relative risks (sRRs) and 95 % CIs for AMI and stroke were estimated using both fixed- and random-effects models. Forest plots were constructed based on random-effects models, and between-study heterogeneity was assessed by graphical inspection and with the Higgins *I*^*2*^ statistic, which describes the percentage of between-study variability in effect estimates attributable to true heterogeneity rather than chance. The Cochran’s χ^*2*^ test of homogeneity and Tau^2^, estimating the between-study variance, are also presented. For studies providing estimates for both monotherapy and combined therapy, we selected the combined-therapy estimate because in clinical practice most patients with T2DM require combined therapy and because it would include more patients (more precision). To explore sources of heterogeneity, we implemented subgroup analyses designed a priori [20], followed by sensitivity analyses in which we excluded studies with high/unclear risk of bias for more than 30 % of the items in the RTI-IB. An additional sensitivity analysis examined the potential impact of the rosiglitazone controversy on the meta-analysis results by grouping the studies according to whether the study period finished before or on 2007, started after 2007, or included 2007, the year in which the first studies showing an increased cardiovascular risk associated with the use of rosiglitazone were published. Publication bias was examined by visual evaluation of funnel plots. The present report follows the MOOSE (Meta-analysis Of Observational Studies in Epidemiology) checklist [25] (see Additional file 1: Table 10e). The present systematic review of published observational studies does not require ethics approval.

## Results and discussion

### Results

**Fig. 1:**
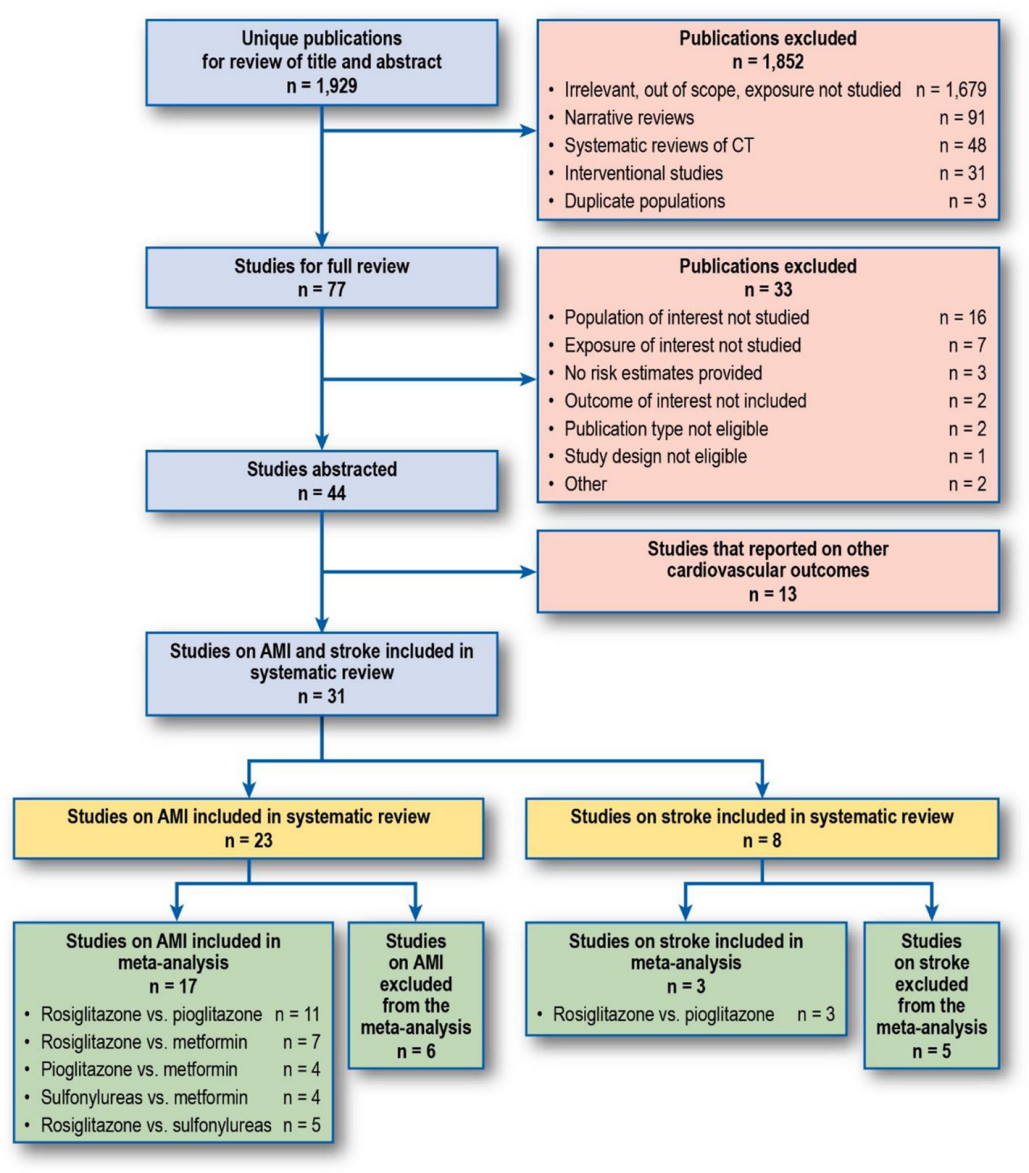
Flow diagram of study identification and selection process. Note: No additional study was identified by checking reference lists of included studies. Some studies contributed to more than one drug-drug comparison

Figure 1 displays the study selection process. From the 44 studies selected for the systematic review, we identified 23 studies evaluating the risk of AMI and 8 studies evaluating the risk of stroke. Of the 23 studies on AMI, 17 contributed to the meta-analysis [26–42], with five drug comparisons: rosiglitazone versus pioglitazone (*n* = 11 studies), rosiglitazone versus metformin (*n* = 7), pioglitazone versus metformin (*n* = 4), rosiglitazone versus sulfonylureas (*n* = 5), and sulfonylureas versus metformin (*n* = 4). Of the 8 studies on stroke, 3 contributed to the meta-analysis for the comparison of rosiglitazone versus pioglitazone [26, 30, 41]. Table 1 displays the list of studies excluded from the meta-analyses for AMI and stroke and the reasons for exclusion.

**Table 1:** Reasons for exclusion of studies

Author, year	Endpoint(s)	Reason for exclusion
Chou, 2011 [52]	Stroke, AMI	The definitions of both stroke and AMI were deemed not eligible. This study included transient ischemic attack in the definition of stroke and stable angina in the definition of AMI.
Azoulay, 2010 [58]	Stroke	Studies excluded due to reference groups combining several medications (e.g., other diabetic drugs)
Habib, 2009 [54]	Stroke, AMI	
Lipscombe, 2007 [57]	AMI	
Dore, 2009 [53]	AMI	Studies reporting comparisons for which inadequate data were available (an inclusion criterion for this meta-analysis was comparison with at least three independent point estimates available)
Hsiao, 2009 [32]	Stroke	
Simpson, 2006 [59]	Stroke	
Horsdal, 2009 [55]	AMI	A more recent study, with updated data, was available (Horsdal, 2011).
Horsdal, 2008 [56]	AMI	

*AMI* indicates acute myocardial infarction

Table 2 displays the main characteristics of the 17 studies evaluating the risk of AMI included in the present meta-analysis. Of the 17 studies, 1 was a case–control study and the rest were cohort studies (3 included a nested case–control analysis). The majority of the studies defined the outcome based on hospitalisations. Most studies included the specific diagnosis code for AMI (i.e., ICD-9 code 410 or ICD-10 codes I21 and I22) in their algorithms to identify cases of AMI; Tzoulaki et al. [38] used Read codes, and Sauer et al. [37] used the Minnesota Heart Study criteria to validate cases of AMI in a field study. Six studies included only first-ever AMI; 11 studies included first-ever AMI plus previous AMI. The number of AMI events in each study ranged from 39 to 15,917. The majority of studies included a broad age range, while a few were restricted to those aged 65 years or older. The definition of exposure varied across studies: 10 studies included only new users of the drugs studied, and the remaining 7 studies included a combination of new and prevalent users. Thirteen studies included patients from North America, 2 studies included European patients, 1 study was conducted in Israel, and 1 in Taiwan. Most of the US studies used claims databases.

**Table 2:** Main characteristics of studies included in the meta-analysis

Reference, source population, study period	Study design, population size, age	Diabetes type 2 population definition	Study endpoints (number of cases)	Case validation	Exposure assessment	Exposure recency	Exposure group(s) vs. reference group (n) A: Comparison(s) contributing to meta-analysis B: Other comparison(s)
Studies included in both meta-analysis endpoints, AMI and stroke							
Bilik, 2010 [26] TRIAD study group USA 2000–2003	Cohort *N* = 2,382 Age ≥ 18 years (patients taking only insulin and aged younger than 30 years were excluded)	First prescription for glitazones. Patients filling prescriptions for more than one TZD were excluded	Non-fatal AMI (ICD-9: 410) (*N* = 39) Non-fatal stroke (ICD-9: 431, 433, 434) (*N* = 32)	None	Prevalent and new users Dispensed prescriptions	Current, continuous use until 90 days after the supply date of their most recently filled prescription duration	A: Rosiglitazone (*n* = 773) vs. pioglitazone (*n* = 711)
Graham, 2010 [30] Medicare, USA 2006–2009	Cohort 227,571 Age ≥ 65 years	First prescriptions for glitazones	Hospitalisation for fatal and non-fatal AMI/ACS/SCHD (ICD-9: 410) (*N* = 1,746) Hospitalisation for fatal and non-fatal acute stroke, ischaemic or haemorrhagic (ICD-9: 430, 431, 433.x1, 434.x1, and 436) (*N* = 1,052)	External PPV for AMI: 89 % – 97 % PPV for stroke: 92 % – 100 %	New users Dispensed prescriptions	Current continuous use, including a gap of no more than 7 days	A: Rosiglitazone (*n* = 67,593) vs. pioglitazone (*n* = 159,978)
Winkelmayer, 2008 [41] Medicare, New Jersey, USA 1999–2005	Cohort 28,361 Age > 65 years	First prescription for a glitazone, regardless of previous treatment with other diabetic drug(s)	Ever and first ever Fatal and non-fatal AMI (ICD-9: not reported ) (*n* = 737) Hospitalisation for a non-fatal stroke: ischaemic or haemorrhagic (ICD-9: 433,434,436) (*N* = 1,869)	External PPV for AMI: 94 % PPV for stroke: 96 %	New users Dispensed prescriptions	Current, continuous use until 60 days after the end of supply date of their most recently filled prescription duration or until switching to other TZD	A: Rosiglitazone (*n* = 14,101) vs. pioglitazone (*n* = 14,260)
Studies included only in the meta-analysis of AMI							
Horsdal, 2011 [31] Danish National Registries 1996–2004	Nested case–control *N* = 101,313 >30 years	Subjects were classified as having T1DM and excluded if they were aged younger than 30 years at the time of their first related prescription or diagnosis and had never received a prescription for an oral glucose–lowering drug. Subjects with T2DM were those with codes for diabetes mellitus who had not received pharmacotherapy, had received prescriptions for oral glucose–lowering drugs, or were aged older than 30 years when they had their first diagnostic code or prescription.	Hospitalisation for AMI (codes not reported) (*N* = 10,616)	External	Prevalent and new users Dispensed prescriptions	At least one prescription of study drug within 90 days before hospitalisation	A: Sulfonylurea monotherapy (*n* = 26,778) vs. metformin monotherapy (*n* = 5,927) B: Sulfonylurea monotherapy (*n* = 26,778) vs. any combination (*n* = 12,425); metformin monotherapy (*n* = 5,927) vs. individual sulfonylurea monotherapy (*n* = 26,778)
Loebstein, 2011 [35] Maccabi Healthcare Services, Israel 2000–2007	Cohort *N* = 15,436 Age, mean (SD): 59.1 (11.4) years	Subjects in the Maccabi diabetes registry with prescriptions for rosiglitazone or metformin for at least 6 months	Hospitalisation for AMI (ICD-9 and Y codes 410XX, Y139XX and Y225XX) (*N* = 645)	None	Prevalent and new users Dispensed prescriptions	Current, continuous use within study period with gaps not longer than 3 months	A: Rosiglitazone monotherapy (*n* = 745) or in combination with metformin (*n* = 2,753) vs. metformin monotherapy (*n* = 11,938) (Formulary restriction allowed to use rosiglitazone only if inadequate control from SU, metformin, or both)
Brownstein, 2010 [27] Partners Healthcare System: Research Patient Data Registry, USA 2000–2006	Cohort *N* = 26,375 Age ≥ 18 years	ICD-9: 250.XX or hemoglobin A1C of at least 6.0 % and at least one record of prescription of an oral diabetes medication as an outpatient or dispensing as an inpatient	Hospitalisation for AMI (ICD-9: 410) (*N* = 1,343)	External PPV: 92 % –94 %	Prevalent and new users Prescriptions issued and dispensed	Current, continuous use within study period with gaps not longer than 6 months	A: Rosiglitazone monotherapy (*n* = 1,879) vs. pioglitazone monotherapy (*n* = 806) or metformin monotherapy (*n* = 12,490) or sulfonylurea monotherapy (*n* = 11,200)
Wertz, 2010 [40] HealthCore Integrated Research Database, USA 2001–2005	Cohort *N* = 36,628 Age ≥ 18 years	First prescription for glitazones	Hospitalisation for AMI (ED visits included) (ICD-9 410.xx) (*n* = 217)	None	New users Dispensed prescriptions	Current use if refill occurred < 1.5 times the days’ supply of the preceding claim for TZD	A: Rosiglitazone (*n* = 14,469) vs. pioglitazone (*n* = 14,469)
Dormuth, 2009 [28] British Columbia Health databases, Canada 1997–2007	Nested case–control *N* = 11,147 Age, mean (SD): 70 (12) years	Subjects with a pharmacy dispensing for metformin, without a dispensing of metformin, other antidiabetic medication, or insulin in the previous 365 days	Hospitalisations for fatal and non-fatal AMI (ICD-9: 410) (*N* = 2,244)	None	New users Dispensed prescriptions	Current use within 90 days of the index date	A: Rosiglitazone (*n* = 462) vs. pioglitazone (*n* = 235) Rosiglitazone (*n* = 462) vs. metformin (*n* = 10,685) Rosiglitazone (*n* = 462) vs. sulfonylurea (*n* = 1,612) Pioglitazone (*n* = 235) vs. metformin (*n* = 10,912) Sulfonylurea (*n* = 1,612) vs. metformin (*n* = 9,535)
Hsiao, 2009 [32] Taiwan Health Insurance Database 2001–2005	Cohort *N* = 473,483 Age, not reported	Subjects with their first ambulatory visit with ICD-9-CM code 250.xx who were prescribed oral blood glucose–lowering agents at least three times. Subjects were excluded if they had T1DM (ICD-9-CM codes 250.x1) or if they had been prescribed only insulin during the study period.	Fatal and non-fatal hospitalisation for AMI (ICD-9: 410.xx and 411.xx) (*N* = 15,917)	None	New users Dispensed prescriptions	Current, continuous use during study period	A: Pioglitazone monotherapy (*n* = 495) or rosiglitazone monotherapy (*n* = 2,093) vs. metformin-based therapy (*n* = 46,444) and vs. SU-based therapy (*n* = 97,651) B: Pioglitazone + SU + metformin (*n* = 9,510) vs. Rosiglitazone + SU + metformin (*n* = 39,962) Pioglitazone + metformin (*n* = 774) vs. rosiglitazone + metformin (*n* = 2,408) Pioglitazone + SU (*n* = 1,231) vs. rosiglitazone + SU (n = 5,141)
Juurlink, 2009 [33] Ontario diabetes database, Canada 2002–2008	Cohort *N* = 39,736 Age ≥ 66 years	First prescription for a glitazone	Hospitalisation for AMI (ICD-10: I20, I21, I22) (*N* = 698)	External PPV ≈ 90 %	New users prescription Dispensed prescriptions	Current use if refill occurred < 1.5 times the days’ supply of the preceding TZD claim	A: Rosiglitazone (*n* = 22,785) vs. pioglitazone (*n* = 16,951)
Tzoulaki, 2009 [38] GPRD, United Kingdom 1990–2005	Cohort *N* = 91,521 Age 35–90 years	One episode of care associated with a clinical or referral event for diabetes and prescriptions for oral blood glucose–lowering treatment	First ever diagnosis of AMI according to Read codes (*N* = 3,588)	External Confirmed 90 % of AMI diagnoses	Prevalent and new users Prescriptions issued	Current, continuous intervals of use within the study period	A: First-generation SU monotherapy (*n* = 6,053) or second-generation SU monotherapy (*n* = 58,095) or rosiglitazone monotherapy (*n* = 8,442) and combination therapy (*n* = 9,640) or pioglitazone including monotherapy and combination therapy (*n* = 3,816) vs. metformin (*n* = 68,181) B: Glibenclamide or gliclazide or glimepiride or glipizide or gliquidone vs. metformin (*n* = 68,181)
Ziyadeh, 2009 [42] i3, USA 2000–2007	Cohort *N* = 95,002 Age ≥ 18 years	Initiators of glitazones	Hospitalisations for fatal and non-fatal AMI (ICD-9: 410.xx) (*N* = 460)	External	New users Dispensed prescriptions	Current, use at index date	A: Rosiglitazone monotherapy (*n* = 47,501) vs. pioglitazone monotherapy (*n* = 47,501)
Koro, 2008 [34] Integrated HealthCore Information Services, USA 1999–2006	Nested case–control *N* = 891,901 Age ≥ 30 years	Subjects with a diagnosis of type 2 diabetes and at least one prescription claim for an antidiabetic agent during their follow-up time available in the database	First-ever hospitalisation for AMI (ICD-9: 410.xx) (*N* = 9,870)	None	Prevalent and new users Dispensed prescriptions	Current use, a prescription in the last 3 months prior to index date	A: Rosiglitazone (*n* = 3,839) vs. pioglitazone (*n* = 3,343)
Walker, 2008 [39] Pharmetrics integrated outcomes database USA 2000–2007	Cohort ≈543,000 Age ≥ 18 years	Subjects were users of rosiglitazone, pioglitazone, metformin, or a sulfonylurea	Hospitalisation for fatal and non-fatal AMI (no codes reported) (*N* = 502)	None	New users Dispensed prescriptions	Current, use at index date	A: Rosiglitazone monotherapy (*n* = 12,440) vs. pioglitazone monotherapy (*n* = 16,302); rosiglitazone monotherapy (*n* = 12,440) or pioglitazone monotherapy (16,302) vs. metformin (*n* = 131,075); rosiglitazone monotherapy (*n* = 12,440) vs. sulfonylurea monotherapy (*n* = 48,376)
Gerrits, 2007 [29] Ingenix Research Database, USA 2003–2006	Cohort *N* = 29,911 Age ≥ 45 years	Subjects with ICD-9 code of 250.xx and a dispensing of pioglitazone or rosiglitazone	Hospitalisation for AMI (ICD-9: 410.xx) (*N* = 375)	External; PPV ≥ 95 %	New users Dispensed prescriptions	Exposure to pioglitazone and rosiglitazone was treated as a unidirectional time-varying covariate; that is, once a patient met the exposure definition, the patient was considered exposed from that point forward, even if the index drug was discontinued	A: Rosiglitazone (*n* = 15,104) vs. pioglitazone (*n* = 14,807)
McAfee, 2007 [36] Ingenix Research Database, USA 2000–2004	Cohort *N* = 33,363 Age ≥ 18 years	Initiators of rosiglitazone, metformin, or a sulfonylurea	Hospitalisation for AMI (ICD-9: 410.xx) (*N* = 226)	External	New users Dispensed prescriptions	Current use during study period. Dispensing of a different study drug or insulin for the monotherapy group (at which time the subject became eligible for a different study cohort); cessation of study drug use alone was not sufficient to end follow-up	A: Rosiglitazone monotherapy (*n* = 8,977) vs. metformin monotherapy (*n* = 8,977) or sulfonylureas monotherapy (*n* = 8,977) B: Rosiglitazone + metformin (*n* = 1,362) or rosiglitazone + sulfonylurea (*n* = 1,362) vs. metformin + sulfonylurea (*n* = 1,362) Rosiglitazone (*n* = 12,874) vs. non-rosiglitzazone drug (*n* = 20,489)
Sauer, 2006 [37] Philadelphia Metropolitan area, USA 1998–2002	Case–control (field study) Controls were community controls selected using random digit dialing *N* = 764 Age 40–75 years	Subjects with T2DM treated with antidiabetic drugs or diet only	First-ever AMI (identified using medical records) (*N* = 113)	AMI validated by Minnesota Heart Survey criteria	Prevalent and new users Interviews	Current, use in the 7 days before index date	A: Sulfonylurea monotherapy (*n* = 158) vs. metformin monotherapy (*n* = 125) B: Sulfonylureas (*n* = 158) or metformin (125) vs. thiazolidinedione (*n* = 26) Sulfonylureas (*n* = 158) vs. thiazolidinediones + sulfonylureas (*n* = 27) Sulfonylureas (*n* = 158) vs. metformin + sulfonylureas (*n* = 102) Metformin (*n* = 125) vs. thiazolidinediones + metformin (*n* = 21)

*ACS* acute coronary syndrome, *AMI* acute myocardial infarction, *ED* emergency department, *GPRD* General Practice Research Database (now the Clinical Practice Research Datalink [CPRD]), *hemoglobin A1C* glycated hemoglobin, *ICD-9* international classification of diseases, 9th revision*, ICD-9-CM*, *International Classification of Diseases, 9th Revision, Clinical Modification;* ICD-10, *International Statistical Classification of Diseases and Related Health Problems, 10th Revision*; PPV, positive predictive value; SCHD, serious coronary heart disease; SD, standard deviation; SU, sulphonylurea(s); T1DM, type 1 diabetes mellitus; *T2DM* type 2 diabetes mellitus, *TZD* thiazolidinedione(s), *USA* United States of America

Note: When it is not indicated that the endpoint is the first ever identified, the study included patients with and without prior history of the study endpoint

Table 2 displays the main characteristics of the 3 studies evaluating the risk of stroke. All were cohort studies comparing rosiglitazone versus pioglitazone [26, 30, 41]. The number of events ranged from 32 to 1,869. Two studies included only elderly subjects that were new users of study medications, while the other study included all subjects aged older than 17 years and both new and prevalent users.

**Fig. 2:**
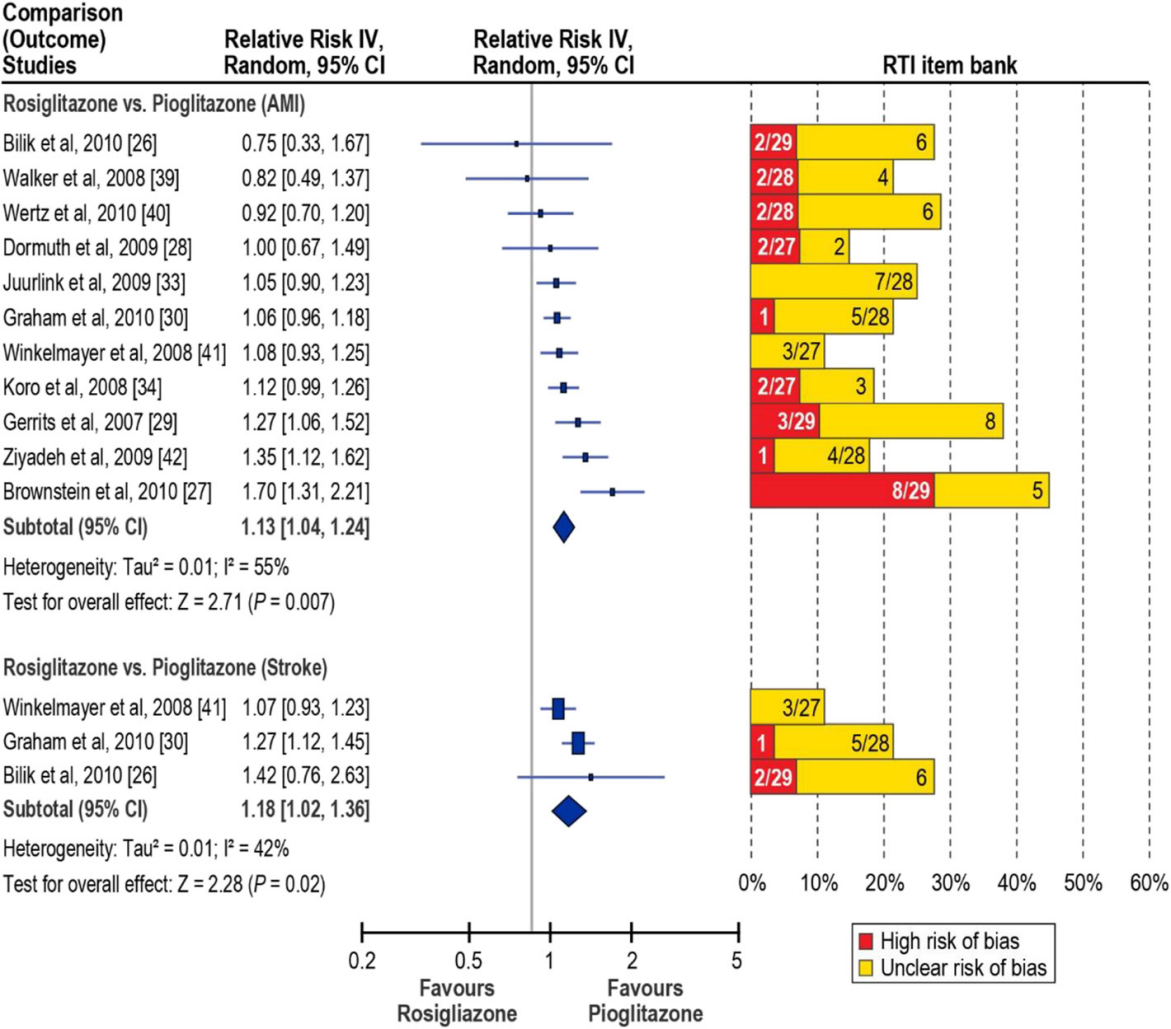
Relative risk of acute myocardial infarction and stroke in rosiglitazone users compared with pioglitazone users. AMI, acute myocardial infarction; IV, inverse variance. Red bars, percentage of items in the RTI item bank indicating high risk of bias; yellow bars, percentage of items at unclear risk of bias. Items with low risk of bias not shown. Denominators indicate the number of items evaluated for each study.

**Fig. 3:**
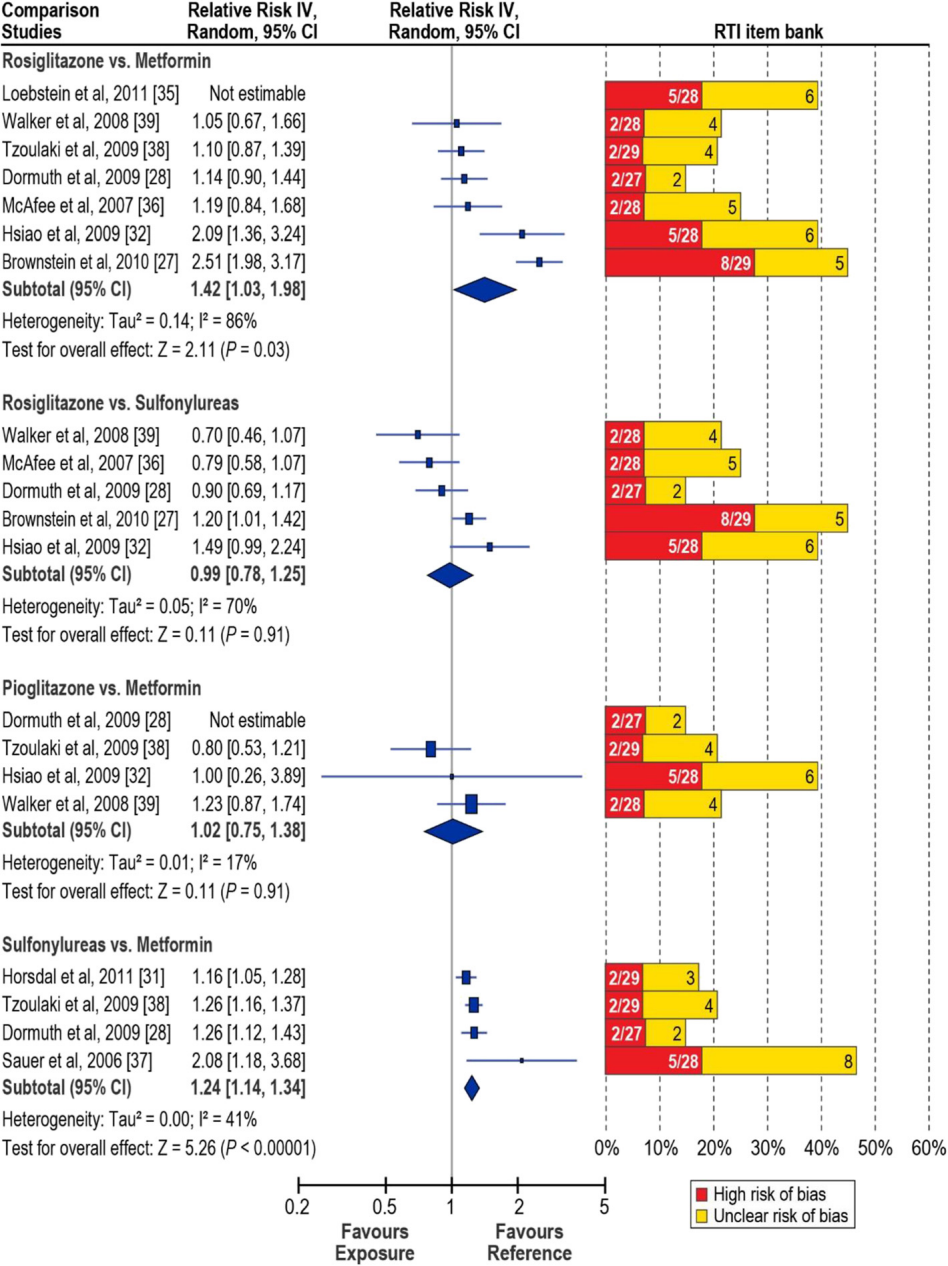
Relative risk of acute myocardial infarction in users of blood glucose–lowering medications. IV, inverse variance. Red bars, percentage of items in the RTI item bank indicating high risk of bias; yellow bars, percentage of items at unclear risk of bias. Items with low risk of bias not shown. Denominators indicate the number of items evaluated for each study. Rosiglitazone versus metformin: Tzoulaki et al. [38] contributed the relative risk reported for combination therapy; for Loebstein et al. [35], the combination-therapy estimate was not estimable. Sulfonylureas versus metformin: Tzoulaki et al. [38] contributed the estimate for second-generation sulfonylureas

In Figs. 2 and 3, the left panel displays the random-effects forest plot for the comparisons included in our meta-analysis. Summary results of study quality assessment with the RTI-IB are provided in the right panel. Detailed results of the Newcastle-Ottawa Scale and the RTI-IB for the 31 studies included in the systematic review are in the Additional file 1. An overview of the experience with and a comparison of the two tools is reported elsewhere [23].

#### Risk of AMI: rosiglitazone versus pioglitazone

The overall sRR (95 % CI) was 1.13 (1.04-1.24), and there was evidence of substantial heterogeneity (*I*^*2*^ = 55 %) (Fig. 2). With the RTI-IB, for AMI, 2 studies had a high/unclear risk of bias for 30 % or more of the items assessed [27, 29]; 2 more studies had a high/unclear risk of bias for 25 to 29 % of the items assessed [26, 40]. For stroke, 1 study had high/unclear risk of bias for 25 % or more of the items assessed [26].

The sensitivity analyses showed that heterogeneity was reduced when restricting the analysis to new users of rosiglitazone and pioglitazone (*I*^*2*^ = 37 %) (Table 3). Even larger reductions in heterogeneity were obtained in the sensitivity analysis excluding the two studies at high/unclear risk of bias for 30 % or more of the items assessed (*I*^*2*^ = 16 %). Heterogeneity was also markedly reduced in the subgroup sensitivity analyses. The sRR did not change much in those analyses and were around 1.10 in most cases, but the 95 % CIs were more conservative; when the two high/unclear-bias studies were removed from the first-ever and prior AMI subgroup and the new users subgroup, the random-effects CIs included the null effect (Table 3).

**Table 3:** Risk of AMI in rosiglitazone users compared with pioglitazone users, overall and subgroup analyses

Study (author, year)	Overall RR (95 % CI)	Subgroup analyses				
		Overall sensitivity analysis^a^	Incident and prevalent cases		New users	
				Sensitivity analysis^a^		Sensitivity analysis^a^
Walker, 2008 [39]	0.82 (0.49–1.37)					
Ziyadeh, 2009 [42]	1.35 (1.12–1.62)					
Brownstein, 2010 [27]	1.70 (1.31–2.21)	Not included		Not included	Not reported	Not reported
Bilik, 2010 [26]	0.75 (0.33–1.67)				Not reported	Not reported
Wertz, 2010 [40]	0.92 (0.70–1.20)					
Dormuth, 2009 [28]	1.00 (0.67–1.49)					
Juurlink, 2009 [33]	1.05 (0.90–1.23)					
Graham, 2010 [30]	1.06 (0.96–1.18)					
Winkelmayer, 2008 [41]	1.08 (0.93–1.25)		Not reported			
Koro, 2008 [34]	1.12 (0.99–1.26)		Not reported		Not reported	Not reported
Gerrits, 2007 [29]	1.27 (1.06–1.52)	Not included		Not included		Not included
Fixed-effects, sRR (95 % CI)	1.12 (1.06–1.18)	1.09 (1.03–1.15)	1.13 (1.06–1.21)	1.08 (1.00–1.16)	1.10 (1.03–1.17)	1.08 (1.01–1.15)
Random-effects, sRR (95 % CI)	1.13 (1.04–1.24)	1.09 (1.02–1.16)	1.14 (1.00–1.30)	1.07 (0.96–1.19)	1.10 (1.01–1.20)	1.08 (0.99–1.18)
Heterogeneity statistics	*τ2* = 0.01 *χ2* = 22.05, df = 10 (*P* = 0.01) *I* ^*2*^ = 55 %	*τ2* = 0.00 *χ2* = 9.56, df = 8 (*P* = 0.30) *I* ^*2*^ = 16 %	*τ2* = 0.02 *χ2* = 21.74, df = 8 (*P* = 0.005) *I* ^*2*^ = 63 %	*τ2* = 0.01 *χ2* = 9.26, df = 6 (*P* = 0.16) *I* ^*2*^ = 35 %	*τ2* = 0.01 *χ2* = 11.06, df = 7 (*P* = 0.14) *I* ^*2*^ = 37 %	*τ2* = 0.00 *χ2* = 8.47, df = 6 (*P* = 0.21) *I* ^*2*^ = 29 %

Not reported indicates that the study did not provide an estimate for that subgroup analysis. Not included indicates that the study was removed as part of the sensitivity analysis

*df* degrees of freedom, *RR* relative risk, *sRR* summary relative risk

^a^Sensitivity analysis: we excluded those studies with a combined high or unclear risk of bias for more than 30 % of the items in the RTI item bank

Of the 11 studies included in the AMI meta-analysis, three reported estimates for monotherapy drug use: one study reported estimates for rosiglitazone and pioglitazone as add-on therapy for metformin users [28], and the rest reported estimates of comparisons in which both rosiglitazone and pioglitazone groups allowed combined treatment with other oral blood glucose–lowering drugs. The monotherapy groups included fewer patients than the combination-therapy groups because very rarely in clinical practice are glitazones prescribed without adjuvant therapy.

#### Risk of stroke: rosiglitazone versus pioglitazone

The sRR (95 % CI) was 1.18 (1.02–1.36); there was no strong evidence of heterogeneity (*I*^*2*^ = 42 %). The meta-analysis evaluating the risk of stroke associated with the use of rosiglitazone compared with pioglitazone included 3 cohort studies of combination therapy (Fig. 2). Two studies used Medicare data and studied patients aged 65 years and older; 1 included patients aged 18 years or older. None reported information on dose or duration.

#### Risk of AMI: rosiglitazone versus metformin

The overall sRR (95 % CI) was 1.42 (1.03–1.98) (Fig. 3), but, there was strong evidence of heterogeneity (*I*^*2*^ = 86 %). With the RTI-IB quality assessment, 2 studies had a high/unclear risk of bias for 30 % or more of the items assessed [27, 32]; and 1 more study had a high/unclear risk of bias for 25 % or more of the items assessed [36].

Subgroup analyses showed that heterogeneity was reduced when restricting the analysis to new users of rosiglitazone and metformin (*I*^*2*^ = 55 %) (Table 4). Even larger reductions in heterogeneity were obtained in the sensitivity analyses excluding the two studies at high/unclear risk of bias for 30 % or more of the items assessed (*I*^*2*^ = 0 %). The sRR decreased in the subgroup analysis (new users) to 1.29 and to 1.13 in the overall sensitivity analysis; the null effect was included in the subgroup analyses random-effects 95 % CI and the overall sensitivity analysis random- and fixed-effects 95 % CIs.

**Table 4:** Risk of AMI in rosiglitazone users compared with metformin users, overall and subgroup analyses

Study (author, year)	Overall RR (95 % CI)		Subgroup analyses	
		Overall sensitivity analysis^a^	New users	
				Sensitivity analysis^a^
Loebstein, 2011 [35]	Not estimable	Not estimable	Not estimable	Not estimable
Walker, 2008 [39]	1.05 (0.67–1.66)			
Tzoulaki, 2009 [38]	1.10 (0.87–1.39)		Not reported	Not reported
Dormuth, 2009 [28]	1.14 (0.90–1.44)			
McAfee, 2007 [36]	1.19 (0.84–1.68)			
Hsiao, 2009 [32]	2.09 (1.36–3.24)	Not Included		Not included
Brownstein, 2010 [27]	2.51 (1.98–3.17)	Not Included	Not reported	Not reported
Fixed-effects, sRR (95 % CI)	1.44 (1.28–1.61)	1.13 (0.98–1.30)	1.24 (1.05–1.47)	1.14 (0.95–1.36)
Random-effects, sRR (95 % CI)	1.42 (1.03–1.98)	1.13 (0.98–1.30)	1.29 (0.99–1.67)	1.14 (0.95–1.36)
Hetrogeneity statistics	*τ* ^*2*^ = 0.14 *χ* ^*2*^ = 36.07, df = 5 (*P* < 0.00001) *I* ^*2*^ = 86 %	*τ* ^*2*^ = 0.00 *χ* ^*2*^ = 0.21, df = 3 (*P* = 0.98) *I* ^*2*^ = 0 %	*τ* ^*2*^ = 0.04 *χ* ^*2*^ = 6.62, df = 3; (*P* = 0.09) *I* ^*2*^ = 55 %	*τ* ^*2*^ = 0.00 *χ* ^*2*^ = 0.17, df = 2; (*P* = 0.92) *I* ^*2*^ = 0 %

*df* degrees of freedom, *RR* relative risk, *sRR* summary relative risk

^a^ Sensitivity analysis: we excluded those studies with a combined high or unclear risk of bias for more than 30 % of the items in the RTI item bank

All 7 studies included in the meta-analysis used a cohort design, but 1 study performed a nested case–control analysis. Only 1 study was restricted to first-ever events. Of the 7 studies, 5 included only new users of the drugs studied, 4 reported estimates only for monotherapy drug use, 1 reported estimates only of comparisons in which the rosiglitazone and pioglitazone groups allowed combination treatment with other oral blood glucose–lowering drugs, and 2 provided estimates for both monotherapy and combination therapy. Only 1 study provided results by exposure duration.

#### Risk of AMI: pioglitazone versus metformin

The sRR (95 % CI) was 1.02 (0.75–1.38), and there was no evidence of significant heterogeneity in a meta-analysis of 3 studies (*I*^*2*^ = 17 %). Four studies were available for this comparison (Fig. 3); however, the 95 % CI reported by Dormuth et al. [28] did not result in an estimable standard error and therefore the study could not be included in the meta-analysis.

#### Risk of AMI: rosiglitazone versus sulfonylureas

The sRR (95 % CI) was 0.99 (0.78–1.25), with strong evidence of heterogeneity for this comparison (*I*^*2*^ = 70 %) (Fig. 3 and Additional file 1: Table 8e). Five studies were available for meta-analysis. In the sensitivity analysis that excluded the high/unclear-bias studies [27, 32], heterogeneity was markedly reduced (*I*^*2*^ = 0 %) and the sRR (95 % CI) decreased to 0.82 (0.69–0.98).

#### Risk of AMI: sulfonylureas versus metformin

The sRR (95 % CI) was 1.24 (1.14–1.34) and evidence of heterogeneity was moderate (*I*^*2*^ = 41 %). Four studies were available for this comparison (Fig. 3, Additional file 1: Table 9e); all analysed sulphonylureas as a group and some also reported on individual agents. Individual agents in the evaluated studies were either first- or second-generation sulphonylureas. For our analysis, we included the reported RR for second-generation sulfonylureas as they were the most frequently used. Three of the four studies [28, 31, 38] provided specific data for the comparison of glyburide versus metformin. When combining those studies (Additional file 1: Table 10e), effect estimates were homogeneous across studies and there was no evidence of heterogeneity (*I*^*2*^ = 0 %). The sRR (95 % CI) was 1.22 (1.14–1.31). Two of the four studies were included in the meta-analysis for only this comparison [31, 37]. One was a field case–control study with an imprecise RR estimate and 30 % or more of the RTI-IB items assessed at high/unclear risk of bias. The other was a nested case–control analysis that included prevalent and new users. Three studies included only first-ever AMI.

No impact on the meta-analysis results was found after grouping the studies according to whether the study period finished before or on 2007, started after 2007, or the study period included 2007 (data not shown).

#### Publication bias

Examination of funnel plots did not suggest publication bias, although the number of studies was small for some comparisons. The funnel plots for studies evaluating the risk of AMI in rosiglitazone users compared with pioglitazone users (*n* = 11), in rosiglitazone users compared with metformin users (*n* = 7), and in rosiglitazone users compared with sulfonylurea users (*n* = 5) are displayed in Additional file 1: Figures 1e, 2e, and 3e.

### Discussion

This systematic review of published observational studies on the risk of AMI associated with glitazones, metformin and sufonylureas in patients with T2DM confirmed that studies in this field are very heterogeneous in exposure definition, comparison drugs, potential for biases secondary to design characteristics, and study results. Therefore, summarising the scientific evidence is challenging. The lack of a common reference medication for evaluation of all potential exposures across studies limited direct comparison of effect estimates. Of the 31 studies included in our systematic literature review, only 20 could contribute to the meta-analysis, 17 for AMI and 3 for stroke.

Our summary effect estimates are compatible with a small increase (around 10%) in the risk of AMI among patients with T2DM using rosiglitazone compared with the risk in those using pioglitazone. However, the degree of heterogeneity present and results of the quality assessment and sensitivity analyses indicate that caution should be used when interpreting this result. Residual confounding might be present in most observational studies; therefore, small increases in risk are difficult to interpret.

Studies in the stroke risk meta-analysis (*n* = 3) had more homogeneous point estimates and quality assessments than studies in the AMI meta-analysis (*n* = 11). Considering that the lower limit of the 95 % CI is very close to 1 and only 3 studies were available for meta-analysis, the observed increase of 18 % in the risk of stroke among patients using rosiglitazone compared with those using pioglitazone should be considered cautiously.

Similar words of caution apply to the comparisons of rosiglitazone versus metformin and rosiglitazone versus sulfonylureas. Although the sRRs indicate a 40 % increase in risk of AMI for the first comparison and no increase in risk for the second comparison, there was strong evidence of heterogeneity for both comparisons, and the sensitivity analyses produced effect estimates closer to the null for the first comparison and further from the null for the second comparison (from sRR of 0.99 to 0.92 in the subgroup analysis and to 0.82 in the sensitivity analysis).

Results reported from the individual studies included in other pioglitazone-versus-metformin and sulfonylureas-versus-metformin comparisons produced more homogeneous results but involved few studies. The sRR for pioglitazone versus metformin suggested no difference in AMI risk for the two drugs, but the other comparison indicated a 24 % higher risk of AMI for sulfonylureas users than for metformin users.

In September 2014, we updated the literature search using the original search terms in PubMed. Two new studies were considered eligible and were reviewed [43, 44]; they compared risk of AMI and of stroke for rosiglitazone and pioglitazone. The updated effect estimates adding these 2 studies remained virtually unchanged: sRR, 1.12 (95 % CI, 1.03–1.21) for AMI and 1.17 (95 % CI, 1.07, 1.27) for stroke.

The results of our meta-analysis are consistent with results of two previous meta-analyses of observational studies evaluating the risk of AMI comparing rosiglitazone and pioglitazone. The meta-analysis of 7 studies conducted by Chen et al. [16] estimated a sRR of 1.17 (95 % CI, 1.04–1.32); *I*^*2*^ = 70 %. Loke et al. [11] conducted a meta-analysis of 13 studies including patients with T2DM, comparing rosiglitazone and pioglitazone safety for AMI, congestive heart failure, and mortality. For AMI, the odds ratio was 1.11 (95 % CI, 1.04–1.18) ; *I*^*2*^ = 16 %. These estimated relative risks are similar to our estimate. Differences in the studies included/excluded between those two meta-analyses and our meta-analysis might explain the differences in statistical heterogeneity.

Three meta-analyses of randomised controlled trials (RCTs) have been published evaluating the safety of rosiglitazone for AMI and stroke and found that rosiglitazone increased the risk of AMI about 40 % compared to control therapies or placebo [12–14]. The results of those meta-analysis were not without controversy [45], and there is evidence that they are sensitive to the statistical method used for pooling and the inclusion/exclusion of different studies [46]. However, the available evidence prompted the European Medicines Agency to withdraw rosiglitazone from the market and the FDA to place severe restrictions on rosiglitazone use [5]. In contrast, meta-analyses indicate a reduced risk of stroke (about 20 %) for rosiglitazone compared to control therapies. Although the 95 % CIs of those estimates include the null effect, the inconsistency of the findings for AMI and stroke is difficult to explain [46]. An independent re-analysis of the RECORD trial data confirmed the original results [9], that rosiglitazone did not increase cardiovascular risk compared to a combination of metformin and sulfonylurea [47]; this has prompted the FDA to ease some restrictions on rosiglitazone use. Another re-analysis of clinical trial data has further questioned the adverse cardiovascular safety profile of rosiglitazone, reporting a hazard ratio of 0.77 for AMI (95 % CI, 0.54–1.10) and of 0.36 (95 % CI, 0.16–0.86) for stroke when comparing rosiglitazone with non-thiazolidinedione use [7].

We did not identify any meta-analysis of RCTs directly comparing rosiglitazone and pioglitazone with AMI as the safety outcome of interest. Two meta-analyses of RCTs comparing pioglitazone with control therapies or placebo found reductions of around 20 % in the risk of AMI or stroke, favouring pioglitazone, with 95 % CIs in both cases including the null effect [48, 49].

For AMI and stroke we could not identify any published meta-analyses of observational studies evaluating the other drug comparisons included in this meta-analysis, i.e., rosiglitazone or pioglitazone versus metformin, rosiglitazone versus sulfonylureas, and sulfonylureas versus metformin. Therefore, the current meta-analysis seems to be the first one to review the results of studies including all those comparisons.

This systematic review and meta-analysis has several strengths. We included in the meta-analysis only studies with a clear definition of the reported comparisons. Our detailed evaluation of the quality of each reviewed study and the sensitivity analyses helped us interpret meta-analysis results of heterogeneous studies combined for the purpose of evaluating the risk of AMI and stroke. Our analyses included drug comparisons relevant to clinicians and not included in previous meta-analyses.

As is true for every meta-analysis of observational studies, the main limitation of this meta-analysis is the heterogeneity in design and conduct of the primary studies. Key drivers of this heterogeneity were the complex array of treatment options, varying severity of diabetes, and varying outcome definitions. The studies that combined medications (e.g., “any other treatment”) as the reference group are of particular concern for this and future meta-analyses for two main reasons. First, “any other treatment” represents different treatments depending on the study period and population, which decreases the applicability and comparability of results. Second, results relative to such reference treatment may not be useful for clinical decision makers who need to choose between specific therapeutic alternatives. The results of this study show that methodological limitations present in the original studies have an important role in explaining the statistical heterogeneity found when combining the individual study results in the meta-analysis. When excluding those studies at high/unclear risk of bias for 30 % or more of the items assessed by the RTI-IB, heterogeneity was largely reduced in most of the comparisons analysed. Overall results in those comparisons were sensitive to the exclusion of the studies more prone to bias, which indicates lack of robustness in results when including such studies in the meta-analysis.

Few of the studies included accounted for severity of diabetes; therefore, confounding by indication could be present in the majority of within-study comparisons for studies in this meta-analysis. Residual confounding might be present in studies that failed to systematically record or adjust for lifestyle factors. Few studies adjusted for socioeconomic status, education, physical activity, or BMI, which can all be associated with both treatment selection and the development of outcomes. These and other methodological limitations of the majority of the studies included in this meta-analysis have been reviewed in recent publications, which support the overall qualitative findings of this study [50, 51]. Since the magnitude of the increased risks was rather small for most of the comparisons, small residual confounding, if present, could explain the small increases in the risk estimates.

Evaluation of the risk of stroke was limited due to the small number of published studies and inclusion of only users of thiazolidinediones. Other limitations of this meta-analysis had to do with the fact that dose and duration effects could not be evaluated for any comparison due to the scarcity of data in the published studies and the fact that the newest oral drugs for diabetes could not be evaluated due to lack of studies.

## Conclusions

In conclusion, sulfonylureas seem to increase the risk of AMI by 24 % compared with metformin. With 3 small studies and a corresponding lack of precision, results of this meta-analysis suggest no difference in the risk of AMI for pioglitazone compared with metformin. The presence of heterogeneity in the meta-analysis results precludes any conclusions on the risk of AMI for the other comparisons evaluated. The quality assessment with the RTI-IB and the sensitivity analyses indicate that statistical heterogeneity might be attributable to the studies that were at the highest risk of bias according to the RTI-IB. Future studies should consider the methodological pitfalls identified in the existing body of evidence. Results from ongoing large multidatabase studies, carefully planned and conducted, are awaited and will help to elucidate the risk of AMI and stroke associated with the use of NIBGLDs.

### Competing interests

RTI Health Solutions employees work on projects funded by pharmaceutical companies including manufacturers of treatments for patients with diabetes. As employees of RTI Health Solutions, Manel Pladevall, Susana Perez-Gutthann, and Cristina Varas-Lorenzo also participate in advisory boards funded by pharmaceutical companies.The authors declare that they have no competing interests

### Authors' contribution

MP, NRG, CVL, AM, and SPG participated in development of the literature search strategy; MP, NRG, and AM abstracted and compiled the data; NRG, BC, AM, MP, and CVL performed the analyses; all authors oversaw design of the study and facilitated interpretation of the findings. MP drafted the manuscript; all coauthors reviewed and revised it critically for important intellectual content and read and approved the final manuscript. All authors had full access to all of the data abstracted from published studies included in this systematic review and take responsibility for the integrity of summarising the data and the accuracy of the meta-analysis.All authors read and approved the final manuscript

## Additional file

Additional file 1: Table 1e. Search Terms for Medline search. **Table 2e**. Newcastle-Ottawa Scale Quality Assessment Results, Individual Case-Control Studies Assessing the Risk of Acute Myocardial Infarction. **Table 3e**. Newcastle-Ottawa Scale Quality Assessment Results, Individual Cohort Studies Assessing the Risk of Acute Myocardial Infarction. **Table 4e**. Newcastle-Ottawa Scale Quality Assessment Results, Individual Case-Control Studies Assessing the Risk of Stroke. **Table 5e**. Newcastle-Ottawa Scale Quality Assessment Results, Individual Cohort Studies Assessing the Risk of Stroke. **Table 6e**. RTI Item Bank Quality Assessment Results: Individual Studies Reporting on the Risk of Acute Myocardial Infarction. **Table 7e**. RTI Item Bank Quality Assessment Results: Individual Studies Reporting on the Risk of Stroke. **Table 8e**. Risk of Acute Myocardial Infarction in Rosiglitazone Users Compared with the Risk in Sulfonylurea Users: Overall, Subgroup (New Users), and Sensitivity Analysis. **Table 9e**. Risk of Acute Myocardial Infarction in Sulfonylurea Users Compared with the Risk in Metformin Users: Overall, and in a Sensitivity Analysis - Summary Relative Risk by Random Effects. **Table 10e**. Risk of Acute Myocardial Infarction in Glyburide Users Compared with the Risk in Metformin Users: Overall - Summary Relative Risk by Random Effects. **Figure 1e**. Funnel Plot of the Relative Risk of Acute Myocardial Infarction for Rosiglitazone Users Compared With Pioglitazone Users (11 Studies). **Figure 2e**. Funnel Plot of the Relative Risk of Acute Myocardial Infarction for Rosiglitazone Users Compared With Metformin Users (7 Studies). **Figure 3e**. Funnel Plot of the Relative Risk of Acute Myocardial Infarction for Rosiglitazone Users Compared With Sulfonylureas Users (5 Studies). (DOCX 278 kb)

## Abbreviations

AMI: Acute myocardial infarction
ESM: Electronic supplementary material
FDA: US Food and Drug Administration
*I*^*2*^: Heterogeneity statistic, interpreted as the percentage of variability on a set of effect size estimates due to heterogeneity between studies rather than sampling error
MOOSE: Meta-analysis of observational studies in epidemiology
NIBGLD: Non-insulin blood glucose–lowering drugs
RCT: Randomised controlled trial
RTI-IB: RTI item bank
sRR: Summary relative risk
T2DM: Type 2 diabetes mellitus
US: United States of America
