# Supplementary material for: Cardiovascular risk associated with the use of glitazones, metformin and sufonylureas: meta-analysis of published observational studies
Source: BMC Cardiovasc Disord. 2016 Jan 15;16:14. doi: 10.1186/s12872-016-0187-5 (PMC4714432; doi:10.1186/s12872-016-0187-5)
Supplement: Additional file 1: Table 1e. — Search Terms for Medline search. Table 2e. Newcastle-Ottawa Scale Quality Assessment Results, Individual Case-Control Studies Assessing the Risk of Acute Myocardial Infarction. Table 3e. Newcastle-Ottawa Scale Quality Assessment Results, Individual Cohort Studies Assessing the Risk of Acute Myocardial Infarction. Table 4e. Newcastle-Ottawa Scale Quality Assessment Results, Individual Case-Control Studies Assessing the Risk of Stroke. Table 5e. Newcastle-Ottawa Scale Quality Assessment Results, Individual Cohort Studies Assessing the Risk of Stroke. Table 6e. RTI Item Bank Quality Assessment Results: Individual Studies Reporting on the Risk of Acute Myocardial Infarction. Table 7e. RTI Item Bank Quality Assessment Results: Individual Studies Reporting on the Risk of Stroke. Table 8e. Risk of Acute Myocardial Infarction in Rosiglitazone Users Compared with the Risk in Sulfonylurea Users: Overall, Subgroup (New Users), and Sensitivity Analysis. Table 9e. Risk of Acute Myocardial Infarction in Sulfonylurea Users Compared with the Risk in Metformin Users: Overall, and in a Sensitivity Analysis - Summary Relative Risk by Random Effects. Table 10e. Risk of Acute Myocardial Infarction in Glyburide Users Compared with the Risk in Metformin Users: Overall - Summary Relative Risk by Random Effects. Figure 1e. Funnel Plot of the Relative Risk of Acute Myocardial Infarction for Rosiglitazone Users Compared With Pioglitazone Users (11 Studies). Figure 2e. Funnel Plot of the Relative Risk of Acute Myocardial Infarction for Rosiglitazone Users Compared With Metformin Users (7 Studies). Figure 3e. Funnel Plot of the Relative Risk of Acute Myocardial Infarction for Rosiglitazone Users Compared With Sulfonylureas Users (5 Studies). (DOCX 278 kb) [file 12872_2016_187_MOESM1_ESM.docx]

Electronic Supplementary Material

Contents

[Table 1e. Search terms for Medline search 2](#_Toc428454278)

[Table 2e. Newcastle-Ottawa Scale quality assessment results: individual case-control studies assessing the risk of acute myocardial infarction 5](#_Toc428454279)

[Table 3e. Newcastle-Ottawa Scale quality assessment results: individual cohort studies assessing the risk of acute myocardial infarction 6](#_Toc428454280)

[Table 4e. Newcastle-Ottawa Scale quality assessment results: individual case-control studies assessing the risk of stroke 8](#_Toc428454281)

[Table 5e. Newcastle-Ottawa Scale quality assessment results: individual cohort studies assessing the risk of stroke 9](#_Toc428454282)

[Table 6e. RTI item bank quality assessment results: individual studies reporting on the risk of acute myocardial infarction 10](#_Toc428454283)

[Table 7e. RTI item bank quality assessment results: individual studies reporting on the risk of stroke 12](#_Toc428454284)

[Table 8e. Risk of acute myocardial infarction in rosiglitazone users compared with the risk in sulfonylurea users: overall, subgroup (new users), and sensitivity analysis 13](#_Toc428454285)

[Table 9e. Risk of acute myocardial infarction in sulfonylurea users compared with the risk in metformin users: overall and in a sensitivity analysis—summary relative risk by random effects 14](#_Toc428454286)

[Table 10e. Risk of acute myocardial infarction in glyburide users compared with the risk in metformin users: overall—summary relative risk by random effects 15](#_Toc428454287)

[Figure 1e. Funnel plot of the relative risk of acute myocardial infarction for rosiglitazone users compared with pioglitazone users (11 studies) 16](#_Toc428454288)

[Figure 2e. Funnel plot of the relative risk of acute myocardial infarction for rosiglitazone users compared with metformin users (7 studies) 17](#_Toc428454289)

[Figure 3e. Funnel plot of the relative risk of acute myocardial infarction for rosiglitazone users compared with sulfonylureas users (5 studies) 18](#_Toc428454290)

Table 1e. Search terms for Medline search

| Search number | Description | Input terms |
| --- | --- | --- |
| #1 | Drug name search | "glyburide"[Mesh] OR "glyburide"[All Fields] OR "glibenclamide"[All Fields] OR "gliclazide"[Mesh] OR "gliclazide"[All Fields] OR "glipizide"[Mesh] OR "glipizide"[All Fields] OR "glimepiride"[Supplementary Concept] OR "glimepiride"[All Fields] OR "tolbutamide"[Mesh] OR "tolbutamide"[All Fields] OR "metformin"[Mesh] OR "metformin"[All Fields] OR "rosiglitazone"[Supplementary Concept] OR "rosiglitazone"[All Fields] OR "pioglitazone"[Supplementary Concept] OR "pioglitazone"[All Fields] OR "acarbose"[Mesh] OR "acarbose"[All Fields] OR "exenatide"[Supplementary Concept] OR "exenatide"[All Fields] OR "liraglutide"[Supplementary Concept] OR "liraglutide"[All Fields] OR "sitagliptin"[Supplementary Concept] OR "sitagliptin"[All Fields] OR "vildagliptin"[Supplementary Concept] OR "vildagliptin"[All Fields] OR "saxagliptin"[Supplementary Concept] OR "saxagliptin"[All Fields] OR "BI 1356"[All Fields] OR "Linaclotide Acetate"[All Fields] OR "linagliptin"[All Fields] OR "repaglinide"[Supplementary Concept] OR "repaglinide"[All Fields] OR "nateglinide"[Supplementary Concept] OR "nateglinide"[All Fields] OR "acetohexamide"[Mesh] OR "acetohexamide"[All Fields] OR "chlorpropamide"[Mesh] OR "chlorpropamide"[All Fields] OR "carbutamide"[Mesh] OR "carbutamide"[All Fields] OR "tolazamide"[Mesh] OR "tolazamide"[All Fields] OR "glyclopyramide"[All Fields] OR "voglibose"[Supplementary Concept] OR "voglibose"[All Fields] OR "miglitol"[Supplementary Concept] OR "miglitol"[All Fields] OR "pramlintide"[Supplementary Concept] OR "pramlintide"[All Fields] OR "alogliptin"[Supplementary Concept] OR "alogliptin"[All Fields] OR "benfluorex"[Supplementary Concept] OR "benfluorex"[All Fields] OR "miglitinide"[All Fields] OR "meglitinide"[Supplementary Concept] OR "meglitinide"[All Fields] |
| #2 | Chemical or pharmacolo­gical class names | "sulfonylurea compounds"[Mesh] OR "sulfonylurea"[All Fields] OR "sulphonylurea"[All Fields] OR "biguanides"[Mesh] OR "biguanide"[All Fields] OR "thiazolidinediones"[Mesh] OR thiazolidinedione*[All Fields] OR "glitazone"[All Fields] OR "glitazones"[All Fields] OR (("alpha-glucosidases"[Mesh] OR "alpha-glucosidase"[All Fields] OR ("alpha"[All Fields] AND "glucosidase"[All Fields]) OR "alpha glucosidase"[All Fields]) AND ("antagonists and inhibitors"[Subheading] OR "antagonist"[All Fields OR "antagonists"[All Fields] OR “inhibitor"[All Fields] OR “inhibitors"[All Fields])) OR (("glucagon-like peptide receptor"[Supplementary Concept] OR "glucagon-like peptide receptor"[All Fields] OR "glucagon like peptide 1 receptor"[All Fields]) AND ("agonists"[Subheading] OR "agonist"[All Fields] OR "agonists"[All Fields])) OR "dipeptidyl-peptidase iv inhibitors"[Mesh] OR ("dipeptidyl-peptidase"[All Fields] AND "iv"[All Fields] AND "inhibitor"[All Fields]) OR "dipeptidyl-peptidase iv inhibitors"[All Fields] OR "dipeptidyl peptidase 4 inhibitors"[All Fields] OR "dipeptidyl-peptidase iv inhibitors"[Pharmacological Action] OR ((("PPAR gamma"[Mesh] OR ("ppar"[All Fields] AND "gamma"[All Fields]) OR "ppar gamma"[All Fields] OR ("peroxisome"[All Fields] AND "proliferator"[All Fields] AND "activated"[All Fields] AND "receptor"[All Fields] AND "gamma"[All Fields]) OR "peroxisome proliferator activated receptor gamma"[All Fields])) AND ("agonists"[Subheading] OR "agonist"[All Fields] OR "agonists"[All Fields])) OR "incretins"[Mesh] OR "incretins"[All Fields] OR "incretins"[Pharmacological Action] |
| #3 | Broader terms | "hypoglycemic agents"[Mesh] OR "hypoglycemic agent"[All Fields] OR "hypoglycemic agents"[All Fields] OR "hypoglycemic agents"[Pharmacological Action] OR "hypoglycaemic agent"[All Fields] OR "hypoglycaemic agents"[All Fields] OR "hypoglycemic drug"[All Fields] OR "hypoglycemic drugs"[All Fields] OR "hypoglycaemic drug"[All Fields] OR "hypoglycaemic drugs"[All Fields] OR "antidiabetic"[All Fields] |
| #4 | OR statement | #1 OR #2 OR #3 |
| #5 | Clinical outcome terms | "Myocardial Infarction"[Mesh] OR myocardial infarction*[Text Word] OR heart attack*[Text Word] OR "Heart Failure"[Mesh] OR heart failure*[Text Word] OR "Arrhythmias, Cardiac"[Mesh] OR arrhythmia*[Text Word] OR "Death, Sudden, Cardiac"[Mesh] OR sudden cardiac death*[Text Word] OR "Brain Ischemia"[Mesh] OR "Intracranial Hemorrhages"[Mesh] OR "Stroke"[Mesh] OR stroke*[Text Word] OR "Cerebrovascular Disorders"[Mesh] OR "cerebrovascular"[Title] OR "cardiovascular"[Title] |
| #6 | Cardiovas­cular mortality outcome terms | "Myocardial Infarction/mortality"[Mesh] OR "Heart Failure/mortality"[Mesh] OR "Arrhythmias, Cardiac/mortality"[Mesh] OR "Death, Sudden, Cardiac/epidemiology"[Mesh] OR "Brain Ischemia/mortality"[Mesh] OR "Intracranial Hemorrhages/mortality"[Mesh] OR "Stroke/mortality"[Mesh] OR "Cerebrovascular Disorders/mortality"[Mesh] OR "Cardiovascular Diseases/mortality"[Mesh] OR sudden cardiac death*[Text Word] OR "cerebrovascular mortality"[Text Word] OR cerebrovascular death*[Text Word] OR "cardiovascular mortality"[Text Word] OR cardiovascular death*[Text Word] |
| #7 | OR statement | #5 OR #6 |
| #8 | Filter terms for observational studies | "Epidemiologic Studies"[Mesh] OR "epidemiologic study"[All Fields] OR "epidemiological study"[All Fields] OR "Cohort Studies"[Mesh] OR ("cohort"[All Fields] AND "study"[All Fields]) OR "cohort study"[All Fields] OR "Risk"[Mesh] OR "Case-Control Studies"[Mesh] OR ("case-control"[All Fields] AND "study"[All Fields]) OR "case-control study"[All Fields] OR ("case"[All Fields] AND "control"[All Fields] AND "study"[All Fields]) OR "case control study"[All Fields] OR "Longitudinal Studies"[Mesh] OR "longitudinal study"[All Fields] OR ("longitudinal"[All Fields] AND "study"[All Fields]) OR "Retrospective Studies"[Mesh] OR ("retrospective"[All Fields] AND "study"[All Fields]) OR "retrospective study"[All Fields] OR "observational"[All Fields] OR "cohort"[All Fields] OR ("case"[All Fields] AND "control"[All Fields]) OR "Cross-Sectional Studies"[Mesh] OR "cross sectional"[All Fields] OR "non-randomized"[All Fields] OR "nonrandomized"[All Fields] OR "non-randomised"[All Fields] OR "nonrandomised"[All Fields] |
| #9 | AND  statement | #4 AND #7 AND #8 |
| #10 | Filter to exclude non- systematic reviews | "Review"[Publication Type] NOT ("Meta-Analysis"[Publication Type] OR "Meta- Analysis as Topic"[Mesh] OR "meta-analysis"[All Fields] OR systematic[sb] OR systematic review*[All Fields] OR "Comparative Effectiveness Research"[Mesh] OR comparative effect*[All Fields]) |
| #11 | Filter for other publication types | "Letter"[Publication Type] OR "Comment"[Publication Type] OR "Randomized Controlled Trial"[Publication Type] OR "Editorial"[Publication Type] |
| #12 | NOT  statement | #9 NOT (#10 OR #11) |
| #13 | Statement to exclude animal studies | "Animals"[Mesh] NOT "Humans"[Mesh] |
| #14 | Final | #12 NOT #13 |

Table 2e. Newcastle-Ottawa Scale quality assessment results: individual case-control studies assessing the risk of acute myocardial infarction

|  |  | Domain and topics | | | | | | | | |  |
| --- | --- | --- | --- | --- | --- | --- | --- | --- | --- | --- | --- |
|  |  | Selection | | | | Comparability | | Exposure | | |  |
| First author | Year | Is the case definition adequate? | Representativeness of the cases | Selection of controls | Definition of controls | Comparability: age and sex | Comparability: additional factors | Ascertainment of exposure | Ascertainment for cases and controls | Non-response rate for cases and controls | Total |
| Horsdal | 2011 | ★ | ★ | ★ | ★ | ★ | ★ | ★ | ★ | ★ | 9 |
| Dormuth | 2009 |  |  | ★ | ★ | ★ | ★ | ★ | ★ |  | 6 |
| Koro | 2008 | ★ | ★ | ★ | ★ | ★ | ★ | ★ | ★ |  | 8 |
| Sauer | 2006 | ★ | ★ | ★ |  | ★ | ★ |  |  | ★ | 6 |
| Dore^a^ | 2009 | ★ |  |  | ★ | ★ | ★ | ★ |  |  | 5 |
| Lipscombe^a^ | 2007 | ★ | ★ | ★ | ★ | ★ | ★ | ★ | ★ |  | 8 |

^a^ These studies were not included in the meta-analysis.

Table 3e. Newcastle-Ottawa Scale quality assessment results: individual cohort studies assessing the risk of acute myocardial infarction

|  |  | Domain and topic | | | | | | | | |  |
| --- | --- | --- | --- | --- | --- | --- | --- | --- | --- | --- | --- |
|  |  | Selection | | | | Comparability | | Outcome | | |  |
| Author | Year | Representativeness of the exposed cohort | Selection of the unexposed cohort | Ascertainment of exposure | Outcome was not present at study start | Comparability: age and sex | Comparability: additional factors | Assessment of outcome | Was follow-up long enough for outcomes to occur? | Adequacy of follow -up of cohorts | Total |
| Loebstein | 2011 | ★ | ★ | ★ |  | ★ | ★ | ★ | ★ | ★ | 8 |
| Bilik | 2010 | ★ | ★ | ★ |  | ★ | ★ | ★ | ★ | ★ | 8 |
| Brownstein | 2010 | ★ | ★ | ★ | ★ | ★ | ★ | ★ | ★ |  | 8 |
| Graham | 2010 | ★ | ★ | ★ | ★ | ★ | ★ | ★ |  | ★ | 8 |
| Wertz | 2010 | ★ | ★ | ★ |  | ★ | ★ | ★ | ★ | ★ | 8 |
| Hsiao | 2009 | ★ | ★ | ★ |  | ★ | ★ | ★ | ★ |  | 7 |
| Juurlink | 2009 | ★ | ★ | ★ |  | ★ | ★ | ★ |  | ★ | 7 |
| Tzoulaki | 2009 | ★ | ★ | ★ |  | ★ | ★ | ★ | ★ | ★ | 8 |
| Ziyadeh | 2009 | ★ | ★ | ★ |  | ★ | ★ | ★ |  | ★ | 7 |
| Walker | 2008 | ★ | ★ | ★ |  | ★ | ★ | ★ | ★ | ★ | 8 |
| Winkelmayer | 2008 | ★ | ★ | ★ | ★ | ★ | ★ | ★ | ★ | ★ | 9 |
| Gerrits | 2007 | ★ | ★ | ★ |  | ★ | ★ | ★ | ★ | ★ | 8 |
| McAfee | 2007 | ★ | ★ | ★ |  | ★ | ★ | ★ | ★ |  | 7 |
| Chou^a^ | 2011 |  | ★ | ★ | ★ | ★ | ★ | ★ | ★ | ★ | 8 |
| Horsdal^a^ | 2008 | ★ | ★ | ★ | ★ | ★ | ★ | ★ | ★ | ★ | 9 |
| Habib^a^ | 2009 | ★ | ★ | ★ |  | ★ | ★ | ★ | ★ | ★ | 8 |
| Horsdal^a^ | 2009 | ★ | ★ | ★ | ★ | ★ | ★ | ★ | ★ | ★ | 9 |

^a^ These studies were not included in the meta-analysis.

Table 4e. Newcastle-Ottawa Scale quality assessment results: individual case-control studies assessing the risk of stroke

|  |  | Domain and topic | | | | | | | | |  |
| --- | --- | --- | --- | --- | --- | --- | --- | --- | --- | --- | --- |
|  |  | Selection | | | | Comparability | | Exposure | | |  |
| First author | Year | Is the case definition adequate? | Representativeness of the cases | Selection of controls | Definition of controls | Comparability: age and sex | Comparability: additional factors | Ascertainment of exposure | Exposure ascertainment for cases and controls | Non-response rate for cases and controls | Total |
| Azoulay^a^ | 2010 | ★ | ★ | ★ | ★ | ★ | ★ | ★ | ★ |  | 8 |

^a^ This study was not included in the meta-analysis.

Table 5e. Newcastle-Ottawa Scale quality assessment results: individual cohort studies assessing the risk of stroke

|  |  | Domain and topic | | | | | | | | |  |
| --- | --- | --- | --- | --- | --- | --- | --- | --- | --- | --- | --- |
|  |  | Selection | | | | Comparability | | Outcome | | |  |
| Author | Year | Representativeness of the exposed cohort | Selection of the unexposed cohort | Ascertainment of exposure | Outcome was not present at study start | Comparability: age and sex | Comparability: additional factors | Assessment of outcome | Was follow-up long enough for outcomes to occur? | Adequacy of follow -up of cohorts | Total |
| Bilik | 2010 | ★ | ★ | ★ |  | ★ | ★ | ★ | ★ | ★ | 8 |
| Graham | 2010 | ★ | ★ | ★ | ★ | ★ | ★ | ★ |  | ★ | 8 |
| Winkelmayer | 2008 | ★ | ★ | ★ | ★ | ★ | ★ | ★ | ★ | ★ | 9 |
| Chou^a^ | 2011 |  | ★ | ★ | ★ | ★ | ★ | ★ | ★ | ★ | 8 |
| Habib^a^ | 2009 | ★ | ★ | ★ |  | ★ | ★ | ★ | ★ | ★ | 8 |
| Hsiao^a^ | 2009 | ★ | ★ | ★ |  | ★ | ★ | ★ | ★ |  | 7 |
| Simpson^a^ | 2006 | ★ | ★ | ★ | ★ | ★ | ★ | ★ | ★ |  | 8 |

^a^ These studies were not included in the meta-analysis.

Table 6e. RTI item bank quality assessment results: individual studies reporting on the risk of acute myocardial infarction

|  |  | Domains and question numbers in each domain | | | | | | | | | | | | | | | | | | | | | | | | | | | | | | |
| --- | --- | --- | --- | --- | --- | --- | --- | --- | --- | --- | --- | --- | --- | --- | --- | --- | --- | --- | --- | --- | --- | --- | --- | --- | --- | --- | --- | --- | --- | --- | --- | --- |
|  |  | Sample definition and selection | | | | | | | Intervention/ exposure | Outcome | Creation of treatment groups | | | Blinding | Soundness of information | | | Follow–up | | | Analysis comparability | | | Analysis outcome | | | Interpretation | Reporting | Added questions | | | |
| Author | Year | 1a | 1b | 2 | 3 | 4 | 5 | 6 | 7 | 8 | 9 | 10 | 11 | 12 | 13 | 14a | 14b | 15 | 16 | 17 | 18 | 19 | 20 | 21 | 22 | 23 | 24 | 25 | 26 | 27 | 28 | 29 |
| Bilik^a^ | 2010 | + | + | + | ? | + | + | - | ? | + | + | + | ? | • | + | + | + | • | ? | ? | + | + | + | + | + | ? | + | + | + | - | + | + |
| Brownstein^a^ | 2010 | + | + | + | - | + | • | + | + | + | + | + | ? | - | ? | - | + | • | + | ? | + | + | + | - | - | ? | - | + | + | ? | - | - |
| Chou | 2011 | + | + | - | + | + | • | - | - | + | + | - | - | • | ? | + | + | • | + | ? | - | - | - | - | - | ? | - | + | + | - | - | - |
| Dore | 2009 | + | + | + | + | ? | • | + | ? | + | + | + | ? | • | + | + | + | • | + | ? | + | + | + | - | + | + | + | + | • | ? | + | + |
| Dormuth^a^ | 2009 | + | + | + | ? | + | • | - | + | + | + | + | ? | • | + | + | + | • | + | • | + | + | + | + | + | + | + | + | + | - | + | + |
| Gerrits^a^ | 2007 | + | + | + | + | + | • | + | ? | + | + | - | ? | • | - | + | + | + | + | ? | + | + | ? | ? | + | ? | ? | + | + | ? | - | + |
| Graham^a^ | 2010 | + | + | + | ? | + | • | + | ? | + | + | + | ? | • | + | + | + | + | - | ? | + | + | + | • | + | + | + | + | + | ? | + | + |
| Habib | 2009 | + | + | + | + | + | • | - | ? | + | + | + | ? | • | + | + | + | + | + | ? | + | + | ? | ? | + | + | + | + | + | - | + | + |
| Horsdal | 2008 | + | + | + | + | + | • | + | + | + | + | + | + | • | + | + | + | + | + | ? | + | + | + | - | - | ? | + | + | + | ? | + | + |
| Horsdal | 2009 | + | + | + | ? | + | • | + | + | + | + | - | + | • | + | + | + | + | + | ? | + | + | + | - | - | ? | + | + | + | ? | + | + |
| Horsdal^a^ | 2011 | + | + | + | + | + | • | + | + | + | + | + | + | • | + | + | + | • | + | ? | + | + | + | • | + | + | + | + | • | + | + | + |
| Hsiao | 2009 | + | + | + | ? | + | • | - | + | + | + | + | + | • | ? | - | + | • | + | ? | + | + | ? | - | + | ? | - | + | - | ? | + | + |
| Juurlink^a^ | 2009 | + | + | + | ? | + | • | + | ? | + | + | + | + | • | ? | ? | + | • | + | ? | + | + | + | ? | + | + | + | + | + | ? | + | + |
| Koro^a^ | 2008 | + | + | + | + | + | • | + | - | + | + | + | - | • | + | + | + | • | + | ? | + | + | + | ? | + | + | + | + | • | ? | + | + |
| Lipscombe | 2007 | + | + | + | + | + | • | + | + | + | + | + | ? | • | + | + | + | • | + | ? | + | + | + | ? | + | + | + | + | • | - | - | + |
| Loebstein^a^ | 2011 | + | + | ? | ? | + | • | + | ? | + | + | + | + | • | + | + | + | • | + | ? | + | - | + | ? | - | + | ? | - | + | - | - | + |
| McAfee^a^ | 2007 | + | + | + | ? | + | • | - | + | + | + | + | ? | • | + | + | + | • | + | ? | + | + | ? | - | + | + | + | + | + | ? | + | + |
| Sauer^a^ | 2006 | + | + | ? | + | + | - | + | + | + | ? | + | ? | - | + | + | + | • | • | ? | + | + | ? | ? | + | ? | - | + | • | ? | - | - |
| Tzoulaki^a^ | 2009 | + | + | + | + | + | • | + | + | + | + | + | + | • | ? | + | + | + | + | ? | + | + | + | ? | + | + | + | + | + | ? | - | - |
| Walker^a^ | 2008 | + | + | + | ? | + | • | - | + | + | + | + | + | • | + | - | + | • | + | ? | + | + | + | ? | + | + | + | + | + | ? | + | + |
| Wertz^a^ | 2010 | + | + | + | ? | + | • | - | + | + | + | + | + | • | + | + | + | • | + | ? | + | + | + | ? | + | ? | ? | + | + | ? | + | - |
| Winkelmayer^a^ | 2008 | + | + | + | ? | + | • | + | ? | + | + | + | ? | • | + | + | + | • | + | • | + | + | + | + | + | + | + | + | + | + | + | + |
| Ziyadeh^a^ | 2009 | + | + | + | + | + | • | + | + | + | + | + | ? | • | + | + | + | • | - | ? | + | + | + | ? | + | + | + | + | + | ? | + | + |

Key: • = not applicable; + = low risk of bias; ­– = high risk of bias; ? = unclear risk of bias.

^a^ Included in meta-analysis.

Table 7e. RTI item bank quality assessment results: individual studies reporting on the risk of stroke

|  |  | Domains and question numbers in each domain | | | | | | | | | | | | | | | | | | | | | | | | | | | | | | |
| --- | --- | --- | --- | --- | --- | --- | --- | --- | --- | --- | --- | --- | --- | --- | --- | --- | --- | --- | --- | --- | --- | --- | --- | --- | --- | --- | --- | --- | --- | --- | --- | --- |
|  |  | Sample definition and selection | | | | | | | Intervention/ exposure | Outcome | Creation of treatment groups | | | Blinding | Soundness of information | | | Follow–up | | | Analysis comparability | | | Analysis outcome | | | Interpretation | Reporting | Added questions | | | |
| Author | Year | 1a | 1b | 2 | 3 | 4 | 5 | 6 | 7 | 8 | 9 | 10 | 11 | 12 | 13 | 14a | 14b | 15 | 16 | 17 | 18 | 19 | 20 | 21 | 22 | 23 | 24 | 25 | 26 | 27 | 28 | 29 |
| Azoulay | 2010 | + | + | + | + | + | • | + | + | + | + | + | + | • | + | + | + | • | + | ? | + | + | + | ? | + | + | ? | + | • | ? | - | + |
| Bilik^a^ | 2010 | + | + | + | ? | + | + | - | ? | + | + | + | ? | • | + | + | + | • | ? | ? | + | + | + | + | + | ? | + | + | + | - | + | + |
| Chou | 2011 | + | + | - | + | + | • | - | - | + | + | - | - | • | ? | + | + | • | + | ? | - | - | - | - | - | ? | - | + | + | - | - | - |
| Graham ^a^ | 2010 | + | + | + | ? | + | • | + | ? | + | + | + | ? | • | + | + | + | + | - | ? | + | + | + | • | + | + | + | + | + | ? | + | + |
| Habib | 2009 | + | + | + | + | + | • | - | ? | + | + | + | ? | • | + | + | + | + | + | ? | + | + | ? | ? | + | + | + | + | + | - | + | + |
| Hsiao | 2009 | + | + | + | ? | + | • | - | + | + | + | + | + | • | ? | - | + | • | + | ? | + | + | ? | - | + | ? | - | + | - | ? | + | + |
| Simpson | 2006 | + | + | + | ? | + | • | + | + | + | + | + | ? | • | + | + | + | • | + | ? | + | + | ? | - | + | + | + | + | + | ? | - | + |
| Winkelmayer^a^ | 2008 | + | + | + | ? | + | • | + | ? | + | + | + | ? | • | + | + | + | • | + | • | + | + | + | + | + | + | + | + | + | + | + | + |

Key: • = not applicable; + = low risk of bias; ­– = high risk of bias; ? = unclear risk of bias.

^a^ Included in meta-analysis.

Table 8e. Risk of acute myocardial infarction in rosiglitazone users compared with the risk in sulfonylurea users: overall, subgroup (new users), and sensitivity analysis

| Study (author, year) | Overall RR (95% CI) | Overall sensitivity analysis^a^ | Subgroup analysis: new users | Sensitivity analysis^a^ |
| --- | --- | --- | --- | --- |
| Walker, 2008 [39] | 0.70 (0.46-1.07) |  |  |  |
| McAfee, 2007 [36] | 0.79 (0.58-1.07) |  |  |  |
| Dormuth, 2009 [28] | 0.90 (0.69-1.17) |  |  |  |
| Brownstein, 2010 [27] | 1.20 (1.01-1.42) | Not included | Not reported | Not reported |
| Hsiao, 2009 [32] | 1.49 (0.99-2.24) | Not included |  | Not included |
|  |  |  |  |  |
| Fixed-effects, sRR (95% CI) | 1.04 (0.92-1.17) | 0.82 (0.69‑0.98) | 0.91 (0.77‑1.07) | 0.82 (0.69‑0.98) |
| Random-effects, sRR (95% CI) | 0.99 (0.78-1.25) | 0.82 (0.69‑0.98) | 0.92 (0.70‑1.22) | 0.82 (0.69‑0.98) |
| Heterogeneity statistics | *τ2* = 0.05  *χ2* = 13.31, df = 4 (*P* = 0.01)  *I^2^* = 70% | *τ2* = 0.00  *χ2* = 1.07, df = 2 (*P* = 0.59)  *I^2^* = 0% | *τ2* = 0.05  *χ2* = 7.91, df = 3 (*P* = 0.05)  *I^2^* = 62% | *τ2* = 0.00  *χ2* = 1.07, df = 2 (*P* = 0.59)  *I^2^* = 0% |

RR, relative risk; sRR, summary relative risk

^a^ Note: For the sensitivity analysis, we excluded those studies with either a high or unclear risk of bias for more than 30% of the items of the RTI item bank.

Table 9e. Risk of acute myocardial infarction in sulfonylurea users compared with the risk in metformin users: overall and in a sensitivity analysis—summary relative risk by random- and fixed-effects

| Study (author, year) | Overall  RR (95% CI) | Sensitivity analysis^a^ |
| --- | --- | --- |
| Horsdal, 2011 [31] | 1.16 (1.05-1.28) |  |
| Tzoulaki, 2009 [38] | 1.26 (1.16-1.37) |  |
| Dormuth, 2009 [28] | 1.26 (1.12-1.43) |  |
| Sauer, 2006 [37] | 2.08 (1.18-3.68) | Not included |
|  |  |  |
| Fixed-effects, sRR (95% CI) | 1.24 (1.17-1.31) | 1.23 (1.16-1.30) |
| Random-effects, sRR (95% CI) | 1.24 (1.14-1.34) | 1.23 (1.16-1.30) |
| Heterogeneity statistics | *τ2* = 0.00  *χ2* = 5.09, df = 3 (P = 0.17)  *I^2^* = 41% | *τ2* = 0.00  *χ2* = 1.80, df = 2 (P = 0.41)  *I^2^* = 0% |

RR, relative risk; sRR, summary relative risk

^a^ For the sensitivity analysis, we excluded those studies with either a high or unclear risk of bias for more than 30% of the items of the RTI item bank.

Table 10e. Risk of acute myocardial infarction in glyburide users compared with the risk in metformin users: overall—summary relative risk by random- and fixed-effects

| Study (author, year) | Overall  RR (95% CI) |
| --- | --- |
| Horsdal, 2011 [31] | 1.17 (1.05-1.30) |
| Tzoulaki, 2009 [38] | 1.23 (1.08-1.40) |
| Dormuth, 2009 [28] | 1.31 (1.13-1.52) |
|  |  |
| Fixed-effects, sRR (95% CI) | 1.22 (1.14-1.31) |
| Random-effects, sRR (95% CI) | 1.22 (1.14-1.31) |
| Heterogeneity statistics | *τ2* = 0.00  *χ2* = 1.48, df = 2 (P = 0.48)  *I^2^* = 0% |

RR, relative risk; sRR, summary relative risk

Figure 1e. Funnel plot of the relative risk of acute myocardial infarction for rosiglitazone users compared with pioglitazone users (11 studies)


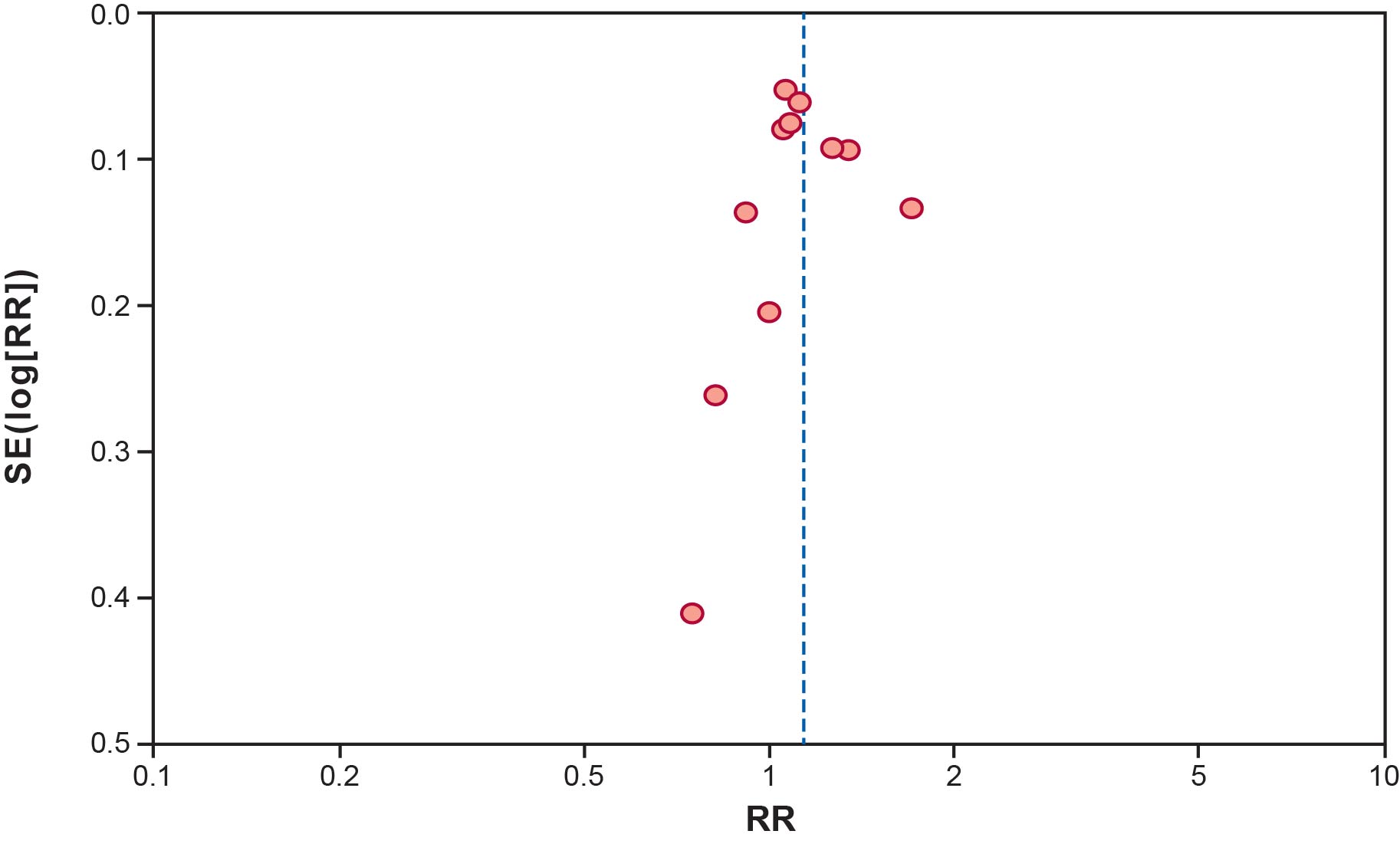


Note: Relative risk (RR) is plotted on the horizontal axis, and an estimate of its precision, SE (log RR), on the vertical axis.

Figure 2e. Funnel plot of the relative risk of acute myocardial infarction for rosiglitazone users compared with metformin users (7 studies)

**
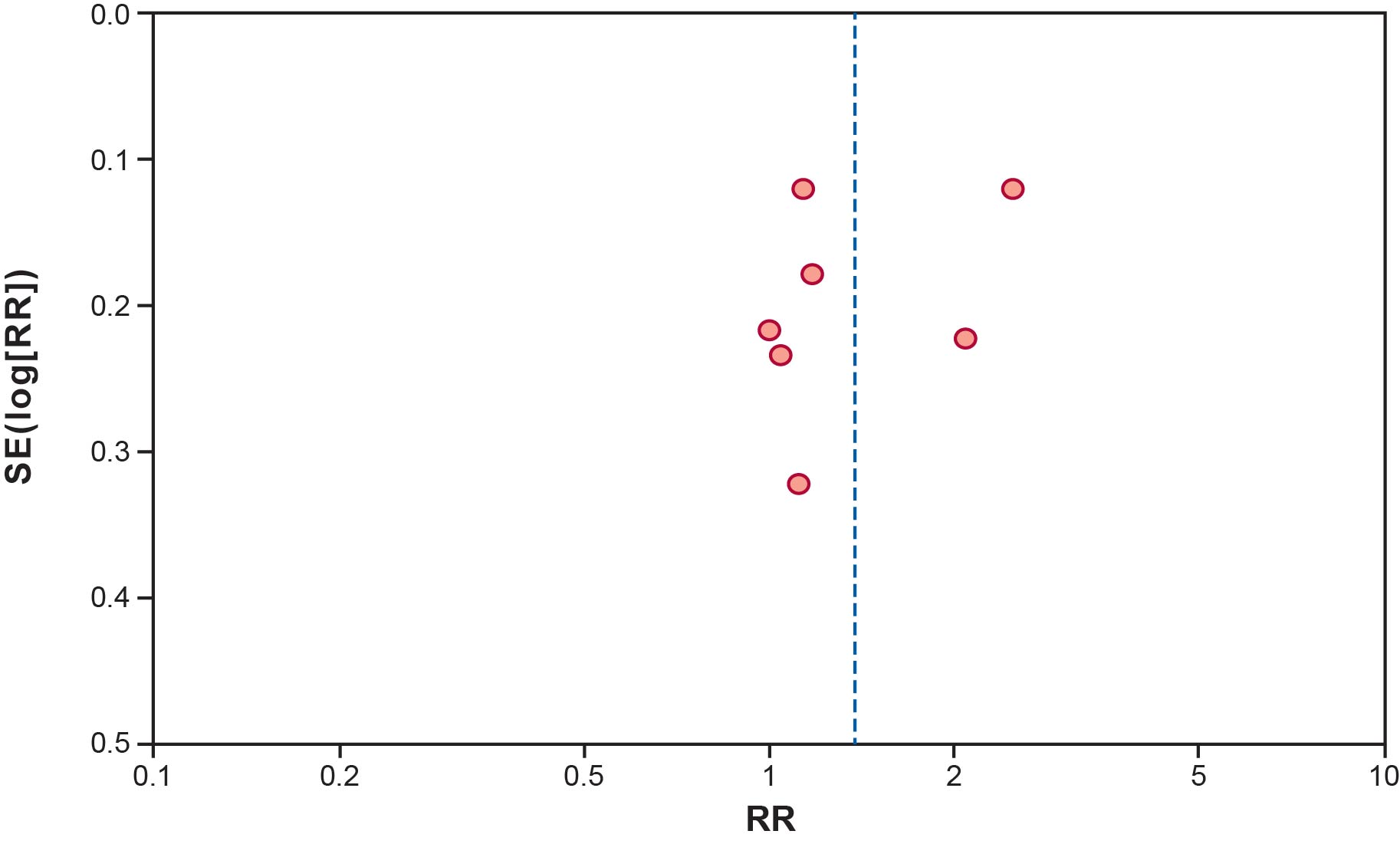
**

Note: Relative risk (RR) is plotted on the horizontal axis, and an estimate of its precision, SE (log RR), on the vertical axis.

Figure 3e. Funnel plot of the relative risk of acute myocardial infarction for rosiglitazone users compared with sulfonylureas users (5 studies)


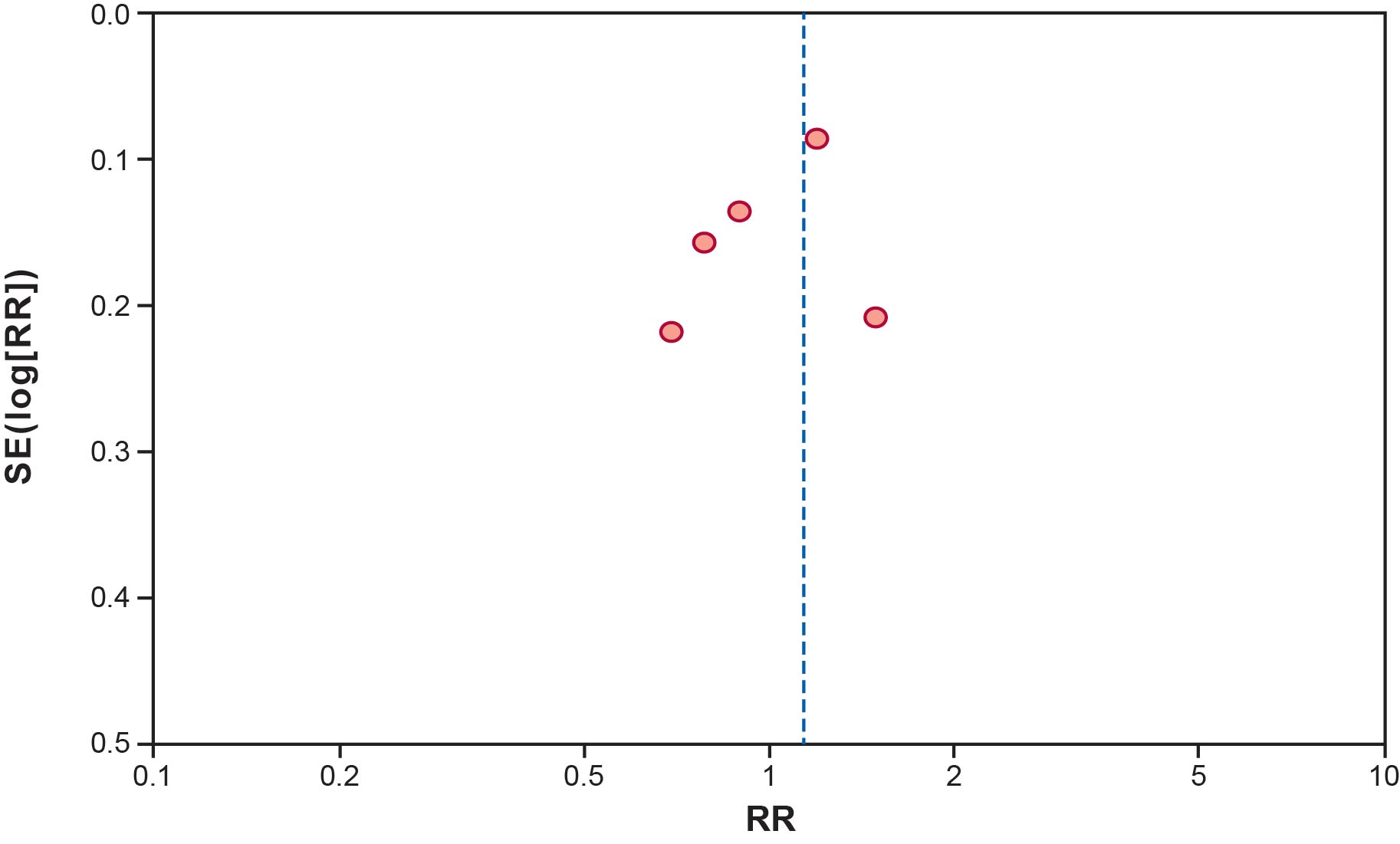


Note: Relative risk (RR) is plotted on the horizontal axis, and an estimate of its precision, SE (log RR), on the vertical axis.
